# UPLC-qTOF-MS Phytochemical Profile and Antiulcer Potential of *Cyperus conglomeratus* Rottb. Alcoholic Extract

**DOI:** 10.3390/molecules25184234

**Published:** 2020-09-15

**Authors:** Abdelsamed I. Elshamy, Abdel Razik H. Farrag, Iriny M. Ayoub, Karam A. Mahdy, Rehab F. Taher, Abd El-Nasser G. EI Gendy, Tarik A. Mohamed, Salim S. Al-Rejaie, Yasser A. EI-Amier, Ahmed M. Abd-EIGawad, Mohamed A. Farag

**Affiliations:** 1 Department of Natural Compounds Chemistry, National Research Center, 33 El Bohouth St., Dokki, Giza 12622, Egypt; rehabfikrytaher@gmail.com; 2 Pathology Department, National Research Centre, 33 El Bohouth St., Dokki, Giza 12622, Egypt; abdelrazik2000@gmail.com; 3 Pharmacognosy Department, Faculty of Pharmacy, Ain Shams University, Cairo 11566, Egypt; irinyayoub@pharma.asu.edu.eg; 4 Medical Biochemistry Department, National Research Centre, 33 El Bohouth St., Dokki, Giza 12622, Egypt; karammahdy64@gmail.com; 5 Medicinal and Aromatic Plants Research Department, National Research Centre, 33 El Bohouth St., Dokki, Giza 12622, Egypt; aggundy_5@yahoo.com; 6 Chemistry of Medicinal Plants Department, National Research Centre, 33 El-Bohouth St., Dokki, Giza 12622, Egypt; tarik.nrc83@yahoo.com; 7 Department of Pharmacology & Toxicology, College of Pharmacy, King Saud University, Riyadh 11451, Saudi Arabia; rejaie@ksu.edu.sa; 8 Department of Botany, Faculty of Science, Mansoura University, Mansoura 35516, Egypt; yasran@mans.edu.eg; 9 Plant Production Department, College of Food & Agriculture Sciences, King Saud University, P.O. Box 2460, Riyadh 11451, Saudi Arabia; 10 Pharmacognosy Department, College of Pharmacy, Cairo University, Kasr el Aini St., Cairo P.B. 11562, Egypt; mfarag73@yahoo.com; 11 Chemistry Department, School of Sciences & Engineering, The American University in Cairo, New Cairo 11835, Egypt

**Keywords:** *Cyperus conglomeratus*, metabolite profiling, UPLC-qTOF-MS, gastroprotective activity, biochemical and histochemical characteristics

## Abstract

*Cyperus* has been commonly used as a multi-use medicinal plant in folk medicine worldwide. The objectives of our study were to determine the different metabolites in the *Cyperus conglomeratus* Rottb. methanol extract, and to assess its in vivo gastroprotective effect in ethanol-induced gastric ulcer model in rats. Serum levels of galactin-3 and TNF-*α* were employed as biochemical markers. To pinpoint for active agents, comprehensive metabolites profiling of extract via UPLC-qTOF-MS/MS was employed. A total of 77 chromatographic peaks were detected, of which 70 were annotated. The detected metabolites were categorized into phenolic acids and their derivatives, flavonoids, stilbenes, aurones, quinones, terpenes, and steroids. Rats were divided into six groups; healthy control, ulcer control, standard drug group, and 25, 50, 100 mg/kg of *C. conglomeratus* treated rats. Pre-treatment with *C. conglomeratus* alcohol extract significantly reduced galactin-3, and TNF-*α* in ethanol-induced ulcer model at 25, 50, and 100 mg/kg. Further histopathological and histochemical studies revealed moderate erosion of superficial epithelium, few infiltrated inflammatory cells, and depletion of gastric tissue glycoprotein in the ulcer group. Treatment with the extract protected the gastric epithelial cells in a dose-dependent manner. It could be concluded that *C. conglomeratus* extract provides significant gastroprotective activity in ethanol-induced gastric ulcer and ought to be included in nutraceuticals in the future for ulcer treatment.

## 1. Introduction

Medicinal plants, throughout history, served as promising resources for natural drugs [1,2]. In these days, medicinal herbs are integral to the resources of safety and potent medicinal drugs due to their bioactive phytochemical components [1]. Among biological actions, anti-ulcerative activities of medicinal plants and their extracts were posed mainly due to their (i) absence and/or low toxicity, (ii) absence and/or low side effects, (iii) high activity and protection, (iv) availability, and (v) low cost [3]. Most of the documented data deduced a relationship among the anti-inflammatory, antioxidant, and antiulcer activities of the herbal plants and their active constituents [1]. Phytochemical components, especially phenolics such as flavonoids, tannins, phenolic acids, stilbenes, quinones, alkaloids, coumarins, as well as steroids, terpenoids/saponins played a significant role in the mechanisms of antiulcerogenic and gastroprotective activities [4,5].

*Cyperus* genus comprising ca. 600 species, is the most widely distributed genus in family Cyperaceae around the world especially in pantropical and tropical areas [5]. *Cyperus* species are widely used in traditional medicines around the world as emollient to treat suckling mothers inflamed breasts, aromatic stomachic in nervous gastralgia, analgesic, diuretic, carminative, stimulant, colic remedy, anthelmintic, and astringent. The previous biological studies on *Cyperus* plants indicated their significant effects including antimicrobial (Sharma et al., 2014), anti-inflammatory [6], hepatoprotective [7], gastroprotective [5], anti-malarial [8], antioxidant [9], and anti-diabetic [10] activities. Several classes of compounds were isolated and reported from *Cyperus* sp. including essential oil [11], quinones [12], flavonoids [5,13], aurones [14,15], sesquiterpenes, steroids [16], and nitrogenous compounds [17].

*Cyperus conglomeratus* Rottb. (Family: Cyperaceae) is an important medicinal plant used in traditional medicine as analgesic, diuretic, stimulant, pectoral, emollient, and anthelmintic [13]. Additionally, few biological activities of the plant alcoholic extract and essential oil were described such as antimicrobial [18], and anti-candidal activities [19]. With regards to metabolites mediating for these effects, several metabolites such as flavonoids [5,12,13], triterpenes, steroids [19], and essential oils [18] were isolated from this plant.

Gastric ulceration results from the imbalance between gastrotoxic agents and protective mechanisms result in acute inflammation. Tumour necrosis factor alpha (TNF-*α*) is one of the major pro-inflammatory cytokines, playing an important role in the production of acute inflammation [20] accompanied by neutrophil infiltration of gastric mucosa [21].

Considering the well-reported medicinal uses of *Cyperus* plants and continuing our search for bioactive products for the treatment of gastric disorders of natural origin [5,6,22], we described herein the first metabolites profile of *C. conglomeratus* alcohol extract via UPLC-qTOF-MS, and its potential gastroprotective activity as monitored using biochemical and histopathological examinations.

## 2. Results and Discussion

### 2.1. Chemical Characterization of C. Conglomeratus Alcohol Extract Using UPLC-Q to f-MS

**Figure 1:**
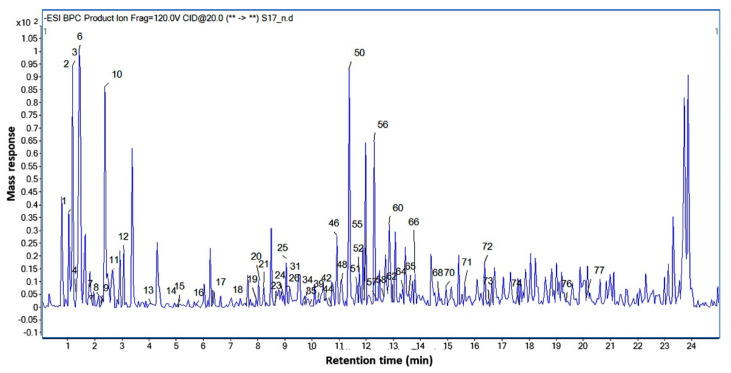
UPLC-qTOF-MS base peak chromatogram of *C. conglomeratus* alcoholic extract in negative ionization mode. Peak numbering follows that stated in Table 1.

A comprehensive metabolite profiling was performed of *C. conglomeratus* alcohol extract used in this bioassays using UPLC-qTOF-MS. A total of 77 chromatographic peaks were detected, of which 70 were annotated (Figure 1). Metabolite assignments were made by comparing retention times and MS data (accurate mass, isotopic distribution, and fragmentation pattern in both negative and positive ionization modes) of detected compounds with those reported in the literature for *Cyperus* species along with searching online public databases. A list of identified compounds with their spectroscopic data was presented in Table 1.

**Figure 2:**
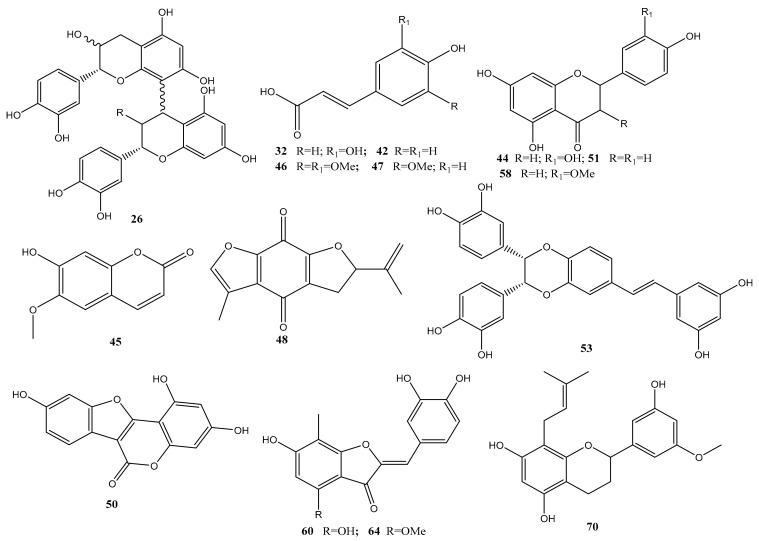
Representative structures of major metabolites identified in *C. conglomeratus* extract.

The identified metabolites belonged to various classes, including organic acids, phenolic acids, cinnamic acid derivatives, flavonoids, stilbenes, aurones, quinones, terpenes, and steroids. Chemical structures of some selected metabolites identified in *C. conglomeratus* extract were illustrated in Figure 2. Phenolic acids represented the most abundant class. Inspection of both negative and positive ionization modes revealed a higher detection level in the negative ionization mode especially for organic acids, phenolic acids, and flavonoids. This is the first detailed metabolites characterization of *C. conglomeratus* using high-resolution UPLC-MS.

#### 2.1.1. Organic Acids

Organic acids were abundant in *C. conglomeratus* extract, eluting early in the chromatogram among which quinic acid (1), citric acid/isocitric acid (6), malic acid (2) and its isomer (7), fumaric acid (8), homocitric acid (12) and its isomers (13, 14) were identified by comparison with published data [5,23,28]. Citric acid and malic acid amounted to the major organic acids.

#### 2.1.2. Hydroxybenzoic Acids

Interpretation of MS spectral data allowed for the identification of several phenolic acids including dihydroxybenzoic acid (**15**) and salicylic acid (**23**), that was proved by the characteristic loss of 44 amu corresponding to the loss of CO_2_ [35]. Asides, syringic acid (peak 40) exhibited a deprotonated molecular ion [M − H]^−^ at *m*/*z* 197.0476 and a subsequent loss of a methoxy group at *m/z* 167 in the MS*^2^* spectrum. Other benzoate derivatives including a methyl ester (**16**) and sugar conjugates (**17**, **21**, **22**) were also identified (Figure S1) in accordance with the literature [24,35].

#### 2.1.3. Hydroxycinnamic Acids

Several hydroxycinnamic acids were annotated in *C. conglomeratus* extract. Similar to hydroxybenzoates, hydroxycinnamic acids also displayed an intense deprotonated molecular ion in the negative ion mode and a subsequent loss of 44 amu corresponding to the loss of CO_2_ from carboxylic functional group [35]. Examples included caffeic acid (**32**) and hydroxycinnamic acid (**42**), hydroxy-dimethoxycinnamic acid (**46**), and ferulic acid (**47**) in comparison with published data [28,36]. Caffeoylquinic acid (**34**) showed a deprotonated molecular ion at *m*/*z* 353.0915 and a production at *m*/*z* 191 correspondences to quinic acid moiety (Figure S2). Peak 35 exhibited a quasi-molecular ion peak at *m*/*z* 371.1001 annotated as syringoylquinic acid. Another syringic acid conjugate was detected in peak 39 [M − H]^−^ *m*/*z* 313.06 and yielding fragment ions at *m*/*z* 197 and 153 correspondences to a syringoyl moiety [C_9_H_9_O_5_]^−^ [35] annotated as syringoylmalic acid (Figure S3).

#### 2.1.4. Cyperaquinones

Peak 48 exhibited [M − H]^−^ at *m/z* 243.0679 of 100% abundance (base peak), suggesting a fully conjugated system recognized as cyperaquinones [29]. A production at *m*/*z* 201 for a loss of 42 amu corresponding to an isopropenyl side chain on the dihydrofuran ring (Figure S4) and was annotated as dihydrocyperaquinone, previously reported in family Cyperaceae [29,37].

#### 2.1.5. Flavonoids

Flavanols, flavanones, and flavonols were detected in *C. conglomeratus* extract and attributed for the majority of peaks in extract. Peaks **18** and **27** were identified as hexahydroxyflavan isomers at [M − H]^−^ at *m*/*z* 305, whereas, peaks **30** and **43** were identified as (epi)catechin [M − H]^−^ at *m*/*z* 289 (Figure S5). Tetrahydroxyflavanones in peaks **44**, **52,** and **59** were identified based on their fragmentation pattern at *m*/*z* 151 and 135 due to RDA fission suggesting a dihydroxy B ring. Likewise, peaks **51** and **61** exhibited [M − H]^-^at *m*/*z* 271 (16 Da less) and were assigned as trihydroxy flavanone. More lypophilic flavanone conjugates were detected in peaks 58 and 68 showing fragment ions at *m*/*z* 285 [M-H-14]^−^, 165 and 135 (RDA fission) confirming the presence of hydroxy and methoxy groups in ring A [34] annotated as trihydroxy-methoxyflavanone isomers. The characteristic loss of 69 amu (prenyl group, –C_5_H_9_) in the MS^2^ spectrum is ascribed to the presence of an isoprenyl group in flavonoids [38] detected in peak **67** and identified as trihydroxy-methoxy-prenylflavone, which was previously isolated from *Cyperus rotundus* [33]. Prenyl flavonoids were previously reported in *C. conglomeratus* [13,34] and are known to exhibit stronger biological effect owing to their improved absorption and or binding to cellular targets. Other detected prenylated flavonoids include trihydroxy-prenylflavan (**69**) and trihydroxymethoxy-prenylflavan (**70**) (Figure S6) in accordance with published data [13,34].

#### 2.1.6. Coumestans/Stilbenes

Peak **50** exhibited a parent ion at *m*/*z* 283.0275 (C_15_H_8_O_6_), displaying successive losses of 28 and 44 amu (Figure S7) and annotated as trihydroxycoumestan in accordance with published data [39]. Likewise, peak **53** exhibited [M − H]^−^ at *m*/*z* 485.1269, C_28_H_22_O_8_^−^ (Figure S8), and identified as longusol C, a stilbene dimer previously isolated from *C. longus* [31].

#### 2.1.7. Aurones

Aurones have been previously reported in various species of Cyperaceae family [40]. Peaks **60** and **66** exhibited [M − H]^−^ at *m*/*z* 299.0589, (MF: C_16_H_12_O_6)_^−^_._ and showing daughter fragments ions at *m*/*z* 284 and 271 correspondences to the loss of a methyl and a carbonyl group, respectively. Aside, abundant productions appearing at *m/z* 165 [A_1_ − H]^−^ and at *m*/*z* 135 ([B_1_ − H]^−^, base peak) (Figure S9) indicating a hydroxylated B-ring, which is characteristic for aurones [41]. Consequently, peaks **60** and **66** were annotated as tetrahydroxy methylated aurone isomers. Likewise, peak **64** exhibited [M − H]^−^ at *m*/*z* 313.0745 (C_17_H_14_O_6_), showing a similar fragmentation behavior to **60** and **66**, with an extra 14 Da (Figure S10) and annotated as trihydroxy-methoxy-methylaurone.

#### 2.1.8. Procyanidins

Procyanidin derivatives were detected in peaks **26** and **38** exhibiting [M − H]^−^ at *m*/*z* 577.1401 and 577.1408, respectively. Characteristic fragment ions of dimeric B type procyanidins were observed at *m*/*z* 451, through the loss of 126 amu by heterocyclic ring fission (HRF) of ring A of the extension unit, epicatechin (Figure S11). In addition, a characteristic loss of 152 amu resulting from the retro-Diels−Alder (RDA) fission was detected at *m*/*z* 425, with a subsequent loss of H_2_O observed at *m*/*z* 407. An MS^2^ fragment ion at *m*/*z* 289 formed by quinonemethide (QM) fission, through the cleavage of the interflavan linkage between the two epicatechin monomers confirmed a procyanidin B type dimer (peaks 26 and 38) [25,26]. Likewise, peak **28** showed a molecular ion peak at *m*/*z* 739.1929 (C_36_H_36_O_17_) with an extra 162 Da exhibiting additional losses of 90 and 120 Da at *m*/*z* 649 and 619 (Figure S12), while displaying similar losses of peaks 26 and 38, suggesting a *C*-hexosylprocyanidin B dimer (**28**) [42].

### 2.2. Gastroprotective Activity

Plants belonging to *Cyperus* genus were reported to encompass various bioactive agents with potential biological effects. We previously reported the anti-inflammatory and gastroprotective activities of different *Cyperus* species extracts such as *C. laevigatus* [6] and *C. alternifolius* [5]. In continuation of our efforts for the evaluation of the biological activity of *Cyperus* extracts, the present study inspected the gastroprotective effects of different doses (25, 50, and 100 mg/kg) of *C. conglomeratus* above ground alcohol extracts on ethanol-induced gastric lesions in rats.

### 2.3. Biochemical Results

In the current study, galactin-3 and TNF-*α* levels in the serum of treated rats were significantly induced in the ethanol-treated group (ulcer group), where it showed 83% increase, compared to the control group. Meanwhile, rats pre-treated with ranitidine prior to oral ethanol administration, showed a significant reduction in both markers exhibiting a reduction of 81% and 79%, respectively, compared to the ethanol-treated group. Nevertheless, significant elevation could still be detected compared to the control group as shown in Table 2.

Rats pre-treated with the extract at a dose of 25 mg/kg reported a significant reduction in TNF-*α* level only relative to both the control and ethanol-treated groups exhibiting a 13.9% reduction relative to the ethanol-treated group. Meanwhile, rats pre-treated with the extract at a dose of 50 or 100 mg/kg reported a significant reduction in galactin-3 and TNF-*α*, compared to both the control and ethanol-treated groups. Pre-treatment with the extract at a dose of 50 mg/kg resulted in a 30.4% and 30% reduction in galactin-3 and TNF-*α* levels, respectively, whereas, rats pre-treated with 100 mg/kg, showed a 97.1% and 39% reduction, respectively, relative to the ethanol-treated group.

Tumor necrosis factor (TNF)-*α* plays an important role in the genesis of gastric mucosal damage [43,44] and its subsequent development [45,46]. Mucus depletion and a constrictive effect on veins and arteries of the gastric mucosa, generating congestion, inflammation, and tissue injury may be attributed to the role of ethanol [47]. *C. conglomeratus* pre-treatment in animals with gastric ulcer significantly reduced the areas of gastric ulcer formation and ulcer index, suggesting the ability of these preparations in protecting gastric ulcer.

A previous study revealed that potential antiulcer drugs can exert their protective effect against gastric lesions through suppression of neutrophil infiltration in gastric tissue [48]. Furthermore, Myeloperoxidase (MPO) is mainly produced by neutrophils and gastric ulcer formation was associated with increases in MPO activity [49]. MPO activity is considered an index of neutrophil infiltration, which can produce many enzymes and free radicals that damage the gastric mucosa, contributing to ulcer development [50].

The increase in the production of TNF-*α* in gastric tissue and serum can be attributed to the necrotizing effects of ethanol [51]. Administration of ethanol may activate the innate immune system leading to the release of pro-inflammatory cytokines, such as TNF-*α* [52]. Previous studies have reported that cytokines such as TNF-*α* play a crucial role in the acute phase inflammation as well as in maintenance and regulation of the severity of gastric ulcers [53]. TNF-*α*, which is an inflammatory cytokine with pleiotropic function, plays a vital role in injury in a variety of tissues including gastric mucosa [54,55]. Moreover, TNF-*α* exhibits synergetic activities to induce the production of NF-kB and other cytokines [56]. The results of this study showed that the levels of TNF-*α* in mice gastric tissue and serum were elevated in the ulcer control group. Administration of *C. conglomeratus* at the three used concentrations at 25, 50, 100 mg/kg significantly reduced the TNF-*α* levels by 13.9%, 30%, and 39%. These results indicated that *C. conglomeratus* ameliorated ethanol-induced gastric ulcers by decreasing pro-inflammatory mediators, such as TNF-*α*.

Galectin-3 can be both anti-apoptotic and pro-apoptotic, and to exhibit anti-inflammatory and pro-inflammatory effects [57,58]. It is highly expressed in the epithelium of the gastrointestinal tract, lungs, and urinary bladder, but is also found in most other tissues [59,60,61]. The present study showed that galactin-3 was significantly increased by 85% in ethanol-treated group compared to the control group. Johannes, et al. [58] suggested that the presence of galectins in serum may result from leakage from tissue, thereby making it a possible disease biomarker. In conclusion, our results showed that levels of galactin-3 and TNF-*α* in serum could be considered as biomarkers of gastric ulcers. These results were further confirmed by histopathological and histochemical studies as described in the following sections.

### 2.4. Histopathological Results

**Figure 3:**
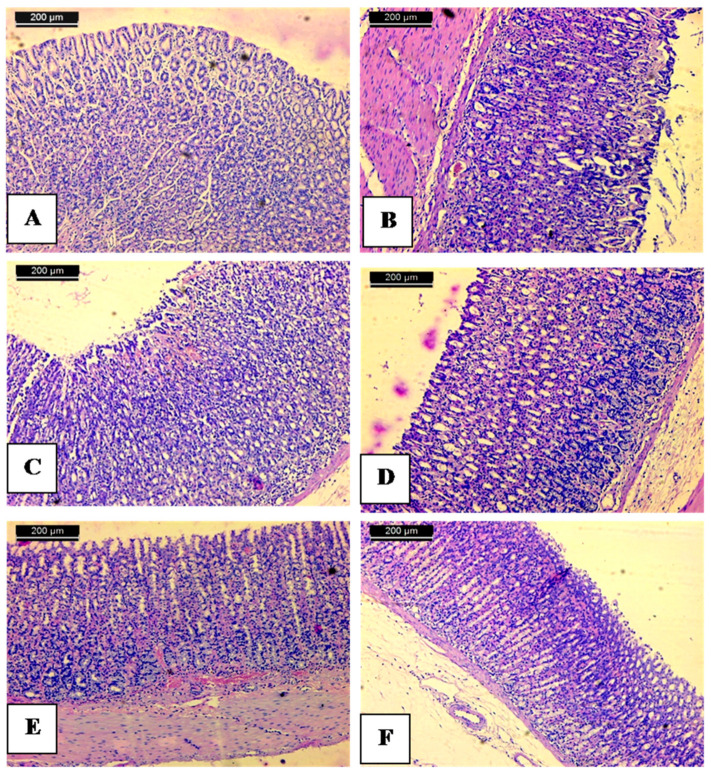
Anti-ulcer activity of 70% alcoholic extract of above ground of *C. conglomerates;* (**A**) normal control showing stomach mucosa with intact surface mucosal epithelium and no lesion appeared; (**B**) stomach mucosa of an ulcer group showing severe disruption of the surface epithelium and necrotic lesions mucosa with inflammatory cells infiltration and hemorrhage as well; (**C**) stomach mucosa of rat treated with 25 mg/kg. b.w of 70% alcoholic *C. conglomeratus* extract showing mild mucosal surface erosion mild disruption to the surface epithelium mucosa with no edema and no leucocytes infiltration of the submucosal layer; (**D**) stomach mucosa of treated with 50 mg/kg. b.w of 70% alcoholic *C. conglomeratus* extract showing mild erosion; (**E**) mucosa of group treated with 100 mg/kg. b.w of 70% alcoholic *C. conglomeratus* extract shows intact surface mucosal epithelium and no lesion appeared; (**F**) mucosa of group treated with Ranitidine showing mild erosion of mucosa. The scale bar length is 200 µm.

To further confirm results revealed from biochemical markers, histopathological examination of injured mucosal tissues versus normal was attempted. The normal control showed the stomach with intact surface mucosal epithelium and without injury (Figure 3A).

The histological study demonstrated that the administration of ethanol caused a disruption of the gastric mucosa coating that protects the gastric mucosa. Stomach mucosa was characterized by the necrosis of epithelial and glandular cells and hemorrhage according to many erythrocytes (Figure 3B). Oral administration of absolute ethanol in the animal model is destructive to stomach tissue, since it penetrates rapidly and easily into the gastric mucosa, producing gastric lesions [62]. Such lesions are characterized by extensive submucosal edema, hemorrhage, desquamation of epithelial cells, and infiltration of inflammatory cells, which are typical characteristics of alcohol injury in humans [63,64]. Ethanol produces necrotic lesions by its direct action on the stomach which reduces factors of defense such as secretion of bicarbonate and the production of mucus [65]. Pre-treatment of ethanol-induced animals with 70% alcoholic extract of above ground of *C. conglomeratus* (25 mg/kg) showed different ulcers with necrosis and hemorrhage in the stomach mucosa (Figure 3C). The group that received *C. conglomeratus* extract (50 mg/kg) displayed a small area of ulcers and hyperaemia (Figure 3D). The animals that received *C. conglomeratus* extract (100 mg/kg) exhibited a dimension in the size of ulcers (Figure 3E). Comparable changes occurred in the group receiving ranitidine as the reference drug. Furthermore, the ranitidine group showed hyperaemia and focal desquamation of epithelial cells (Figure 3F).

Metabolites profiling of *C. conglomeratus* extract revealed its richness in flavonoids, aurones, tannins, phenolic acids, stilbenes, quinones, alkaloids, coumarins, as well as steroids/terpenoids. These phytoconstituents, particularly flavonoids and phenolic acids were previously established to be among the possible cytoprotective agents involved in reducing gastric ulcer [66,67].

### 2.5. Histochemical Results

**Figure 4:**
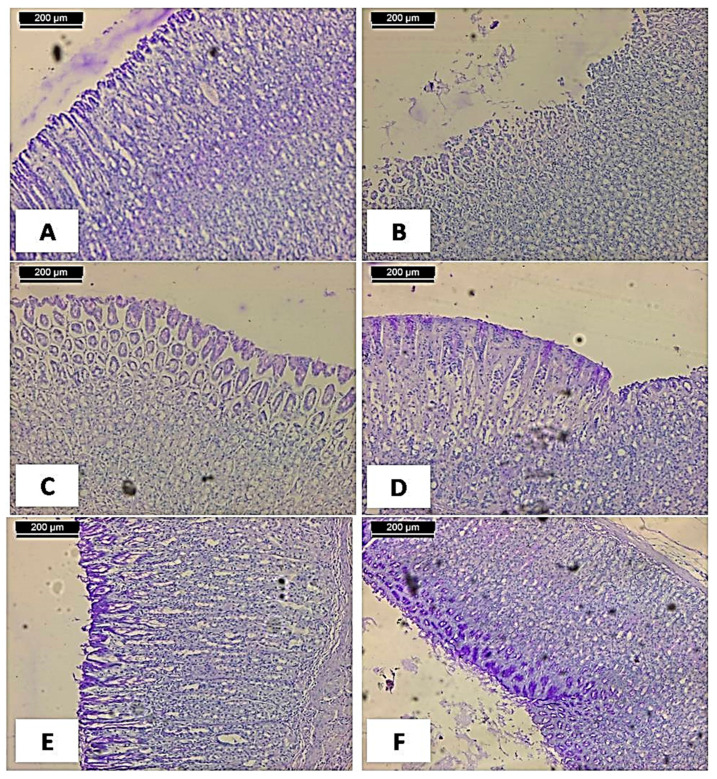
The effects of 70% alcoholic extract of above ground of *C. conglomeratus* on gastric tissue glycoprotein in ethanol-induced gastric ulcers in rats. (**A**) Normal control showing no accumulation of the magenta color in the mucosal cell layer; (**B**) stomach mucosa of ulcer group showing no accumulation of the magenta color in the mucosal cell layer; (**C**, **D**, and **E**, respectively): 25, 50, and 100 mg/kg. b.w of 70% alcoholic *C. conglomeratus* extract showing an increase in magenta color in the mucosal cell layer compared to the ulcerated group in a dose-dependent manner; (**F**) rat treated with Ranitidine showing an increase in magenta color in the mucosal cell layer compared to the ulcerated group (PAS stain, scale bar; 200 µm).

The effects of 70% alcoholic extract of above ground of *C. conglomeratus* on gastric tissue glycoprotein in ethanol-induced gastric ulcers in rats were illustrated in Figure 4. The periodic acid Schiff (PAS) staining was higher in the control group as manifested by the accumulation of the magenta color that indicates the glycoprotein content in the mucosal cell layer (Figure 4A). On the other hand, the magenta staining decreased or diminished and was observed to be not plentiful in the gastric mucosa of the ulcerated group (Figure 4A). At the same time, using 25, 50, or 100 mg/kg of 70% alcoholic *C. conglomeratus* extract, an increase in magenta color in the mucosal cell layer compared to the ulcerated group was observed in a dose-dependent manner (Figure 4C–E). In the same context, ranitidine treated group showed an increase in magenta color in the mucosal cell layer compared to the ulcerated group (Figure 4F).

The observed strong magenta color in the apical epithelial cells of the control group indicated the presence of glycoprotein in the gastric mucosa. This intense coloration in the PAS stain was not observed in the ulcer group. This observation is in agreement with previous reports [68,69]. The same sharp intense increase in magenta coloration of the glandular epithelia was also observed in the ulcer pretreated groups using 25, 50, or 100 mg/kg of 70% alcoholic extract of *C. conglomeratus* as dose-dependent. Our results confirmed the antiulcer activity of *C. conglomeratus* that could be attributed to the highly polyphenolic contents such as flavonoids, phenolic acids, and stilbenes in addition to other metabolites such as terpenes and steroids. The ulcers were combined mainly with excretion of the factors of inflammation including TNF-*α* [70] that caused the migration of polymorphonuclear neutrophil in addition to adhesion molecules up-adjustment in the endothelial and neutrophil cells [44]. All these ulceration factors decreased the production of the mucosal NO, which play an effective role as gastroprotective mediator, and thus the cytokines such as interleukins [71]. The NO releasing caused several ulcer activators such as sub-mucosal arterioles vasodilation, and thus an increase of the flow of blood that followed with the destroyed ability of gastric acid capacity and the toxins that were removed [72].

The gastroprotective role of flavonoids and phenolic compounds were described via their anti-inflammatory actions [6], and decreasing of levels of TNF-α [5]. The role of polyphenolic metabolites as potent antioxidant agents, especially flavonoids, is well reported, especially when increasing the gastroprotection activity of these compounds against lesions by decreasing lipoperoxides levels and increasing glutathione peroxidase enzyme activity. Zakaria et al. [73] and Abdelwahab*,* et al. [74] reported that polyphenolics are capable of activating the defense of the mucosal system by stimulating the secretion of gastric mucus as well as scavenging free radicals and ROS in EtOH-induced gastric ulcer in rats. Additionally, these phenolic compounds increase gastric mucosa formation via decreasing ulcerogenic lesions and thus inhibition of pepsinogen production and acid mucosal secretion [75]. Since flavonoids, phenolic acids, and aurones are potent scavengers of free and lipid radicals due to the presence of active oxygenated sites, these compounds might interact synergistically and/or partly as ulcer protectors [76].

Phenolic compounds displayed gastroprotective effects via modulation of oxidative stress and stimulation of prostaglandin E2 production [77]. Among the major phenolic identified in *C. conglomeratus* to mediate for the gastroprotective effects include dihydroxy benzoic acid (**15**) to inhibit *ex*-*vivo* leukotriene C4 (LTC4) formation in gastric mucosa, a mediator of gastric mucosal damage [78].

Interestingly, an abundance of hydroxycinnamic acids and their derivatives in extract (Table 1) exhibited additionally marked anti-inflammatory activity via downregulating cytokines responsible for mediating inflammation (TNF-α, IL1*β*, IL-6, and IL-8) [79]. In addition, these compounds inhibit the NF-κβ pathway concerned with the expression of inflammatory mediators [80]. Ferulic acid (**47**) demonstrated mucosal membrane protection causing a significant reduction in ethanol-induced gastric ulcers. Quinic acid (**1**) has been shown to exhibit antioxidant and anti-neuroinflammatory activities [81]. Caffeoylquinic acid derivatives show anti-inflammatory activity by inhibiting the pro-inflammatory cytokines TNF-*α* and IL-1*β* [82].

Asides from phenolic acids amounting to the major secondary metabolite class in *C. conglomeratus*, flavonoids exhibit cytoprotective effects and are well-recognized for their anti-ulcer activity [5]. The anti-ulcer activity of flavonoids has been proposed via several mechanisms; increasing prostaglandins mucosal content, decreasing histamine secretion, scavenging free radicals, increasing vascular perfusion, and reducing leukocyte adhesion [83]. Examples of which include naringenin, a trihydroxy flavanone (**51**) that accelerates the healing of gastric ulcers [47], and catechin (**30**), which inhibits TNF-*α* release by blocking NF-kB activation [84]. Nevertheless, isolation of single pure compounds is necessary to provide a conclusive understanding of the antiulcer activity observed herein.

## 3. Materials and Methods

### 3.1. Plant Material Collection and Preparation of Extract

The above-ground parts of *C. conglomeratus* were collected from sand dunes in the Northern Mediterranean coast, near Alshihabiyyah, Baltim, Kafr Elsheikh Governorate, Egypt (31°35′12.47″ N, 31° 8′26.62″ E) in early June 2018A voucher sample (No: CYCO/018-01086) was deposited at the Herbarium of Faculty of Science, Mansoura University.

The air-dried powder of the above-ground parts (450 g) of *C. conglomeratus* was extracted with 70% MeOH (3 L) at room temperature and filtered. The extraction was repeated three times. The extract was collected, dried under reduced pressure to afford black gum (18.2 g), and stored in the refrigerator at 4 °C until further chemical and biological analyses.

### 3.2. High-Resolution Ultra-Performance Liquid Chromatography-Mass Spectrometry Analysis (UPLC-ESI–Qtof-MS)

The air-dried powder of the above-ground parts of *C. conglomeratus* (1 g) was extracted with a mixture of 3:7 (*v*/*v*) of H_2_O-MeOH for 1 h over ultrasonic bath (Branson ultrasonic corporation, Danbury, CT, USA). The extract mixture was filtered and centrifuged for 15 min, and then the clear supernatant extract liquid was applied for analysis via UPLC-ESI–Qtof-MS.

Chromatographic separation was performed on an ACQUITY UPLC system (Waters, Milford, MA, USA) equipped with a HSS T3 column (100 × 1.0 mm, particle size 1.8 µm; Waters) followed the procedure described in Maamoun*,* et al. [85]. The analysis was carried out using a binary gradient elution system at a flow rate of 150 µL/min: 0 to 1 min, isocratic 95% A (water/formic acid, 99.9/0.1 [*v*/*v*]), 5% B (acetonitrile/formic acid, 99.9/0.1 [*v*/*v*]); 1 to 16 min, linear from 5% to 95% B; 16 to 18 min then isocratic 95% B; 18 to 20 min, and finally, isocratic 5% B. Full loop injection volume (3.1 µL) was used. The system was coupled to a 6540Agilent Ultra-High-Definition (UHD) Accurate-Mass Q-TOFLC/MS (Agilent Technologies, Santa Clara, CA, USA) equipped with an ESI interface. Data acquisition (2.5 Hz) in profile mode was governed by MassHunter Workstation software (version B.04.00, Agilent technologies, Santa Clara, CA, USA). The spectra were acquired in negative and positive ionization modes, over a mass-to-charge (*m*/*z*) range from 70 to 1100. The detection window was set to 100 ppm. Characterization of compounds was performed by the generation of the candidate formula with a mass accuracy limit of 10 ppm, and also considering RT, MS2 data, and reference literature.

### 3.3. Experimental Animals

Healthy female Wistar rats weighing 140 to 170 g were purchased from the animal laboratory of the National Research Centre (NRC) and used for pharmacological studies. Prior to the study, rats were acclimatized for few days under normal environmental conditions (12 h dark/12 h light cycle, temperature 20 to 22 °C, relative humidity 40% to 60%) and fed on rodent pellets and water ad libitum. The study was conducted according to the rules of the ethics committee of the National Research Centre and in accordance with the Guide for the Care and Use of Laboratory Animals of the National Institutes of Health in compliance with the guidelines from the Canadian Council on Animal Care (approval no: 18–151).

### 3.4. Ulcer Induction

The previous study deduced the safety of *C. conglomeratus* alcoholic extract with LD_50_ > 4000 mg/kg [19]. The ulcer was induced experimentally by a single oral administration of 1 mL/kg/rat of absolute alcohol according to the method described by Park, et al. [63] with some modifications. Prior to the start of this experiment, rats were fasted for 18 h but allowed free access to water until the beginning of this experiment.

### 3.5. Experimental Design and Animal Grouping

Rats were randomly divided into six groups of six animals per group as follows: Group I: control rats); Group II: ethanol ulcerated group according to Elshamy, et al. [22]; Group III: ulcerated rats pre-treated with 30 mg/kg ranitidine as a reference drug; Group IV: ulcerated rats pre-treated with 25 mg/kg of the extract; Group V: ethanol ulcerated rats pretreated with 50 mg/kg of extract. Group VI: ulcerated rats pre-treated with 100 mg/kg of extract. Ranitidine was purchased from Pharco Co. (Alexandria, Egypt) and prepared as suspensions in 1% Tween 80 in sterile distilled water.

### 3.6. Galactin-3 and TNF- α Determination

Serum galectin-3 and TNF-*α* levels were determined by enzyme-linked immunosorbent assay technique, using kits purchased from Sun Red Biotechnology (Shanghai, China). The operational processes were measured in accordance with the instructions of the kit. The experiment was carried out three times and all results were expressed as means ± standard error. The statistical significance of differences for each parameter between groups was evaluated by one-way ANOVA, followed by the LSD test, and the significance level was set at *p* < 0.05. The analysis was performed using SPSS 19.0 (SPSS Inc., Chicago, IL, USA).

### 3.7. Histopathology

After measuring the ulcer area, small pieces of stomachs from each group were embedded in paraffin wax. Sections of 5 µm thick were cut in a microtome and mounted on glass slides using standard techniques. After staining the tissues with hematoxylin-eosin stains, the slides were viewed under a light microscope equipped for photography [86].

### 3.8. Gastric Mucosal Glycoprotein Evaluation

To evaluate gastric mucosal glycoprotein, sections (5 µm) of each stomach were stained with periodic acid Schiff (PAS) in order to take clear observation of gastric epithelial mucus secretion and to indicate a better assessment of any changes in glycoprotein [87].

## 4. Conclusions

The current study provides the first insights into the gastroprotective potential of *C. conglomeratus* alcohol extract in ethanol-induced gastric ulcer in rats. Comprehensive phytochemical profiling of *C. conglomeratus* extract was performed for the first time herein. A total of 70 compounds were identified, belonging to several classes of secondary metabolites including phenolic acids, flavonoids, stilbenes, aurones, quinones, terpenes, and steroids. These bioactive compounds, especially phenolic acids and flavonoids, are likely to mediate the cytoprotective effects involved in reducing gastric ulcer. *C. conglomeratus* extract exerted promising anti-inflammatory actions via suppressing the serum levels of TNF-*α* and galactin-3 in a dose-dependent manner. The antiulcer activity of *C. conglomeratus* was further confirmed by histopathological, histochemical examinations as evidenced by amelioration of inflammation and preservation of the gastric mucosa against ethanol deleterious effects. Hence, *C. conglomeratus* can be introduced as a promising gastroprotective natural remedy and to be further incorporated in nutraceuticals. The detailed metabolite profile presented herein presents potential chemical markers to be used for standardization and QC analysis of this herbal drug. The next logical step would be to identify exact bioactive phenolics in that complex extract using classical isolation in parallel to biological testing of isolated chemicals to be more conclusive.

## Figures and Tables

**Table 1:** Identified metabolites in *C. conglomeratus* alcoholic extract via UPLC-qTOF-MS.

No	Name	Formula	RT	[M − H] *m*/*z*	Exact Mass	Diff (ppm)	Ms Fragmentation	Class	Ref.
1.	Quinic acid	C_7_H_12_O_6_	1.038	191.0584	192.0657	−9.16	131, 127, 85	Organic acid	[5]
2.	Malic acid	C_4_H_6_O_5_	1.129	133.0162	134.0235	0.05	115	Organic acid	[23]
3.	Unidentified	C_30_H_30_O_9_	1.147	533.1781		7.33	473, 377, 191, 133		
4.	Tetrahydroxypentanoic acid	C_5_H_10_O_6_	1.312	165.0423	166.0495	−10.85	75	Organic acid	
5.	Unidentified	-	1.404	195.8125			162.84, 61.98		
6.	Citric acid/isocitric acid	C_6_H_8_O_7_	1.449	191.0222	192.0295	−12.96	159, 111, 87, 67	Organic acid	[5]
7.	Malic acid	C_4_H_6_O_5_	2.089	133.0158	134.0232	−11.99	115	Organic acid	[23]
8.	Fumaric acid	C_4_H_4_O_4_	2.226	115.0052	116.0124	−12.83	99, 87,72, 71	Organic acid	
9.	Unidentified	C_23_H_10_O_2_	2.272	317.0588	318.0654	8.42	225, 197, 150		
10.	Unidentified	-	2.363	195.813		-	162.84, 80.97		
11.	Leucine-hexose	C_12_H_23_ NO_7_	2.683	292.1419		−6.02	257, 130, 84	Amino acid	
12.	Homocitric acid	C_7_H_10_O_7_	3.094	205.0378	206.0449	−10.92	179, 161, 126, 111, 85	Organic acid	
13.	Homocitric acid (Isomer)	C_7_H_10_O_7_	4.054	205.0376	206.0449	−10.92	161, 126, 111, 87	Organic acid	
14.	Homocitric acid (Isomer)	C_7_H_10_O_7_	5.014	205.037	206.0439	−6.06	161, 126, 111, 87	Organic acid	
15.	Dihydroxybenzoic acid	C_7_H_6_O_4_	5.06	153.0213	154.0283	−11.03	109	Phenolic acid	[5]
16.	Dihydroxybenzoic acid methyl ester	C_8_H_8_O_4_	5.745	167.0363	168.0435	−7.4	151, 125, 123, 108, 81	Phenolic acid derivative	
17.	Dihydroxy benzoic acid-*O*-hexoside	C_13_H_16_O_9_	6.339	315.0751	316.0821	−8.37	153, 97	Phenolic acid derivative	
18.	Hexahydroxyflavan (gallocatechin)	C_15_H_14_O_7_	7.527	305.0702	306.0771	−10.45	179, 167, 125	Flavanol	
19.	Dihydroxy benzoic acid isomer	C_7_H_6_O_4_	7.938	153.0214	154.0286	−13.03	109	Phenolic acid	[5]
20.	Unidentified	C_11_H_20_O_8_	8.075	279.1104	280.1174	−5.74	117, 89		
21.	Dihydroxy benzoic acid methyl ester hexoside	C_14_H_18_O_9_	8.167	329.09	330.0972	−6.35	270 [M – H − COOCH_3_]^−^, 167 [M – H − 162]^−^, 153, 125, 108	Phenolic glycoside	[24]
22.	*O*-hexosyl-*O*-methyl-myo-inositol-dihydroxy benzoic acid	C_20_H_30_O_14_	8.258	493.1601	494.166	−4.85	331 [M − H − 162]^−^, 293, 243, 209, 167, 137 [M − H − 162 − 194]^−^, 89	Phenolic glycoside	
23.	Salicylic acid	C_7_H_6_O_3_	8.624	137.0257	138.0329	−8.64	93 [–COO]^−^	Phenolic acid	
24.	Piscidic Acid (p-hydroxybenzoyl) tartaric acid	C_11_H_12_O_7_	8.944	255.0529	256.0613	−11.6	165, 149, 135	Organic acid	
25.	Benzoyl tartaric acid	C_11_H_10_O_7_	9.035	253.0378	254.0449	−8.69	195, 123	Organic acid	
26.	Procyanidin B dimer	C_30_H_26_O_12_	9.172	577.1401	578.148	−9.52	451, 425, 407, 289	Proanthocyanidin	[25,26]
27.	Hexahydroxyflavan	C_15_H_14_O_7_	9.218	305.0699	306.0767	−8.93	179, 167, 125	Flavanol	
28.	C-hexosylprocyanidin B dimer	C_36_H_36_O_17_	9.263	739.1929	740.2011	−7.92	649 [M − H − 90]^−^, 619 [M − H − 120]^−^, 587 [M − H − 152]^−^ RDA, 449 [M − H − 289]^−^ QM, 289	Proanthocyanidin	
29.	Unidentified	C_17_H_22_O_11_	9.309	401.1124	402.1163	−0.14	313, 267, 193, 151		
30.	(epi)catechin	C_15_H_14_O_6_	9.355	289.0742	290.0813	−7.67	245, 205, 203, 179	Flavanol	
31.	Dimethoxyhomophthalic acid	C_11_H_12_O_6_	9.492	239.0587	240.0655	−8.85	195 [M − H − COO]^−^, 179 [M − H − 2 OCH_3_]^−^, 149 [M − H − 2 COOH]^−^, 133, 107, 87	Organic acid	
32.	Caffeic acid	C_9_H_8_O_4_	9.583	179.0366	180.0439	−9.09	135	Phenolic acid	
33.	Hydroxymethoxycinnamaldehyde	C_10_H_10_O_3_	9.629	177.0574	178.0647	−9.42	134	Aldehyde	
34.	*O*-Caffeoylquinic acid	C_16_H_18_O_9_	9.72	353.0915	354.0977	−7.38	191	Phenolic acid	
35.	*O*-Syringoylquinic acid	C_16_H_20_O_10_	9.903	371.1001	372.106	−0.86	121	Phenolic acid	
36.	Caffeoquinone	C_9_H_6_O_4_	10.04	177.0213	178.0287	−11.65	149 [M − H − CO]^−^, 135, 133 [M − H − CO_2_]^−^,105 [M − H − COOCH_2_]^−^, 93	Phenolic acid	[27]
37.	Unknown	C_14_H_24_O_10_	10.086	351.1332	352.1398	−8.01	293, 249, 191, 173, 133 (Malic acid)	Organic acid	
38.	Procyanidin B dimer	C_30_H_26_O_12_	10.177	577.1408	578.1483	0.08	451 [M − H − 126]^−^, 425 [M − H − 152]^−^, 407 [M − H − 152 − 18]^−^, 289 [M − H − 288]^−^	Proanthocyanidin	[25,26]
39.	Syringoylmalic acid	C_13_H_14_O_9_	10.223	313.06	314.0669	−10.03	197, 153, 121	Phenolic acid	
40.	Syringic acid	C_9_H_10_O_5_	10.269	197.0476	198.0547	−9.35	167 [M − H − 2CH_3_]^−^, 121, 78	Phenolic acid	
41.	Dihydroxyhomophthalic acid dimethyl ester	C_11_H_12_O_6_	10.451	239.0586	240.0657	−9.48	195 [M − H − COO]^−^, 179 [M − H − 2 OCH_3_]^−^, 149 [M − H − 2 COOH]^−^, 133, 107, 87	Organic acid	
42.	Hydroxycinnamic acid	C_9_H_8_O_3_	10.497	163.0421	164.0496	−13.56	119	Phenolic acid	[5]
43.	(epi)-Catechin	C_15_H_14_O_6_	10.634	289.0738	290.0808	−6.07	245, 205, 203, 179	Flavanol	
44.	Eriodictyol	C_15_H_12_O_6_	10.726	287.0587	288.0661	−9.31	151, 135	Flavanone	
45.	Scopoletin	C_10_H_8_O_4_	10.817	191.0349	192.0422	0.5	176, 148, 134,107	Coumarin	[17]
46.	Hydroxydimethoxycinnamic acid	C_11_H_12_O_5_	10.908	223.0635	224.0708	−10.52	163 [M − H − 2 OCH_3_]^−^, 133, 117, 91	Phenolic acid	[28]
47.	Ferulic acid	C_10_H_10_O_4_	10.954	193.0526	194.0599	−10.33	149,134, 107	Phenolic acid	
48.	Dihydrocyperaquinone	C_14_H_12_O_4_	11.091	243.0679	244.075	−5.81	201 [M − H − 42]^−^, 159		[29]
49.	Caffeoquinone isomer	C_9_H_6_O_4_	11.274	177.0205	178.0279	−7.02	133, 89	Phenolic acid	
50.	Trihydroxycoumestan	C_15_H_8_O_6_	11.365	283.0275	284.035	−10.13	255 [M − H − CO]^−^, 239 [M − H − COO]^−^, 133	Coumestan	[30]
51.	Trihydroxyflavanone	C_15_H_12_O_5_	11.639	271.0637	272.071	−9.26	151, 119	Flavanone	
52.	Tetrahydroxyflavanone	C_15_H_12_O_6_	11.731	287.0583	288.0651	−5.97	151, 135	Flavanone	
53.	Longusol C	C_28_H_22_O_8_	11.777	485.1269	486.133	−3.11	375, 241, 177, 109	Stilbene dimer	[31]
54.	Hydroxy-methoxycoumarin	C_10_H_8_O_4_	11.822	191.0334	192.0409	7.11	176,148, 121,104	Coumarin	
55.	Trihydroxycinnamic acid dimethyl ether	C_11_H_12_O_5_	11.914	223.0634	224.0708	−10.51	164, 146, 133, 117, 91	Phenolic acid	
56.	Tetrahydroxyflavone (Luteolin)	C_15_H_10_O_6_	12.279	285.0439	286.0512	−12.14	199, 151, 135, 133 [M − C_8_H_6_O_2_ − H_2_O]^−^, 107	Flavone	[32]
57.	Dihydroxy-dimethoxyflavone (3′,4′-Dimethoxy luteolin)	C_17_H_14_O_6_	12.371	313.074	314.0812	−7.03	298, 284, 254,	Flavone	
58.	Trihydroxy-methoxyflavanone (Hesperitin)	C_16_H_14_O_6_	12.69	301.0742	302.0814	−7.77	285, 165, 135	Flavanone	
59.	Tetrahydroxyflavanone	C_15_H_12_O_6_	12.736	287.0589	288.0661	−9.41	151, 135	Flavanone	
60.	Tetrahydroxymethylaurone	C_16_H_12_O_6_	12.827	299.0589	300.0662	−9.48	284 [M − H − CH_3_]^−^, 271 [M − H − CO]^−^, 179,165, 151, 135	Aurone	[15]
61.	Trihydroxyflavanone	C_15_H_12_O_5_	12.873	271.0641	272.0714	−10.69	191, 151, 135	Flavanone	
62.	Hydroxymethoxycinnamaldehyde	C_10_H_10_O_3_	12.965	177.0574	178.0644	−8.07	162 [M − H − CH_3_]^−^, 117 [M − H − OCH_3_]^−^, 91 (M − H − OCH_3_ − CO)	Aldehyde	
63.	Trihydroxy-octadecadienoic acid	C_18_H_32_O_5_	13.193	327.2209	328.2283	−10.04	299 [M − H − 28]^−^, 285, 229, 171, 85	Fatty acid	[5]
64.	Trihydroxy-methoxy methyl aurone	C_17_H_14_O_6_	13.33	313.0745	314.0817	-8.56	298 [M − CH_3_]^−^, 283 [M − H − CHO), 270, 164, 149, 136, 121	Aurone	[14,15]
65.	Trihydroxy-octadecenoic acid	C_18_H_34_O_5_	13.559	329.2364	330.2439	−9.8	311 [M − H − 18]^−^, 285 [M − H − 44]^−^, 229, 211, 171	Fatty acid	
66.	Tetrahydroxymethylaurone isomer	C_16_H_12_O_6_	13.787	299.059	300.066	−8.79	284 [M − H − CH_3_]^−^, 271 [M − H − CO]^−^, 165, 151, 135	Aurone	[15]
67.	Trihydroxymethoxyprenyl isoflavone	C_21_H_20_O_6_	13.924	367.1226	368.1297	−9.96	352 [M − H − CH_3_]^−^, 298 [M − H − C_5_H_9_]^−^, 269 [M − H − C_5_H_9_ − CHO]^−^, 165, 151, 135	Isolavone	[33]
68.	Tetrahydroxyflavanone methyl ether	C_16_H_14_O_6_	14.655	301.0748	302.082	−9.82	285, 165, 135	Flavanone	
69.	Trihydroxy-prenylflavan	C_20_H_22_O_4_	14.747	325.1481	326.1549	−9.61	311, 283, 249,241, 203, 183, 163, 145, 121, 109	Flavan	
70.	Trihydroxymethoxy prenylflavan	C_21_H_24_O_5_	14.929	355.1583	356.1658	−9.68	321, 295 [M − OCH_3_]^−^, 267, 219, 149, 135, 97	Flavan	[13,34]
71.	Unknown steroid	C_32_H_44_O_9_	15.66	571.2934	572.3006	−3.59	525, 315, 241, 153	Steroid	
72.	Unknown steroid	C_26_H_34_O_4_	16.392	409.2387	410.2454	0.86	315, 153	Steroid	
73.	Triterpene-Unidentified	C_30_H_38_O_4_	16.574	461.2701	462.2779	−2.03	279, 153	Triterpene	
74.	Unknown steroid	C_27_H_36_O_4_	17.625	423.2552	424.2622	-2.07	255, 153	Steroid	
75.	Triterpene-Unidentified	C_30_H_38_O_4_	17.717	461.2704	462.2776	−1.36	279, 181	Triterpene	
76.	Unknown steroid	C_26_H_34_O_4_	19.362	409.2407	410.2476	−4.68	279, 153, 79	Steroid	
77.	Triterpene-Unidentified	C_30_H_38_O_4_	20.093	461.2714	462.2787	−3.73	279, 181, 125	Triterpene	

**Table 2:** Effects of oral administration of *C. conglomeratus* alcoholic extract on blood Galactin-3 (ng/mL) and TNF-*α* (Pg/mL) levels in ethanol-induced gastric ulcer in rats.

Parameters	Galactin-3 (ng/mL)		TNF-α Pg/mL	
Groups	Mean ± S.E	% Change	Mean ± S.E	% Change
Control	1.7 ± 0.14	0	30.61 ± 1.07	0
Ethanol (1 mL)	11.10 ± 0.85 ^a^	+85 ^a^	212.71 ± 3.71 ^a^	+85 ^a^
Ranitidine + Ethanol	2.06 ± 0.13 ^a b^	−81.4 ^b^	44.25 ± 1.77 ^a,b^	−79 ^b^
Ex (25 mg) + Ethanol	11.01 ± 0.46 ^a^	−0.8 ^b^	183.15 ± 5.93 ^a,b^	−13.9 ^b^
Ex (50 mg) + Ethanol	7.75 ± 0.29 ^a b^	−30.4 ^b^	148.83 ± 5.75 ^a,b^	−30 ^b^
Ex (100 mg) + Ethanol	5.63 ± 0.37 ^a b^	−97.1 ^b^	128.98 ± 6.07 ^a,b^	−39 ^b^

All data presented as means ± standard error, ^a^ significantly different at *p* < 0.05 and % of change as compared with the control group, ^b^ significantly different at *p* < 0.05 and % of change as compared with ulcer group.

## Supplementary Materials

The following are available online, Figure S1–Figure S12: Negative ESI tandem mass spectra of peak 21, 34, 39, 48, 30, 70, 50, 53, 66, 64, 26, and 28.

## References

[1] Asnaashari S., Dastmalchi S., Javadzadeh Y. (2018). Gastroprotective effects of herbal medicines (roots). Int. J. Food Prop..

[2] Park J.U., Kang J.H., Rahman M.A.A., Hussain A., Cho J.S., Lee Y.I. (2019). Gastroprotective effects of plants extracts on gastric mucosal injury in experimental sprague-dawley rats. BioMed Res. Int..

[3] Bansal V.K., Goel R.K. (2012). Gastroprotective effect of *Acacia nilotica* young seedless pod extract: Role of polyphenolic constituents. Asian Pac. J. Trop. Med..

[4] de Lira Mota K.S., Dias G.E.N., Pinto M.E.F., Luiz-Ferreira Â., Monteiro Souza-Brito A.R., Hiruma-Lima C.A., Barbosa-Filho J.M., Batista L.M. (2009). Flavonoids with gastroprotective activity. Molecules.

[5] Farrag A.R.H., Abdallah H.M.I., Khattab A.R., Elshamy A.I., Gendy A., Mohamed T.A., Farag M.A., Efferth T., Hegazy M.F. (2019). Antiulcer activity of *Cyperus alternifolius* in relation to its UPLC-MS metabolite fingerprint: A mechanistic study. Phytomedicine.

[6] Elshamy A.I., El-Shazly M., Yassine Y.M., El-Bana M.A., Farrag A.-R., Nassar M.I., Singab A.N., Noji M., Umeyama A. (2017). Phenolic constituents, anti-inflammatory and antidiabetic activities of *Cyperus laevigatus* L.. Pharmacogn. J..

[7] Kumar S.S., Mishra S. (2005). Hepatoprotective activity of rhizomes of *Cyperus rotundus* Linn against carbon tetrachloride-induced hepatotoxicity. Indian J. Pharm. Sci..

[8] Thebtaranonth C., Thebtaranonth Y., Wanauppathamkul S., Yuthavong Y. (1995). Antimalarial sesquiterpenes from tubers of *Cyperus rotundus*: Structure of 10, 12-peroxycalamenene, a sesquiterpene endoperoxide. Phytochemistry.

[9] Kumar K.H., Razack S., Nallamuthu I., Khanum F. (2014). Phytochemical analysis and biological properties of *Cyperus rotundus* L.. Ind. Crops Prod..

[10] Raut N.A., Gaikwad N.J. (2006). Antidiabetic activity of hydro-ethanolic extract of *Cyperus rotundus* in alloxan induced diabetes in rats. Fitoterapia.

[11] Nassar M.I., Yassine Y.M., Elshamy A.I., El-Beih A.A., El-Shazly M., Singab A.N.B. (2015). Essential oil and antimicrobial activity of aerial parts of *Cyperus leavigatus* L. (Family: Cyperaceae). J. Essent. Oil Bear. Plant..

[12] Nassar M.I., Abdel-Razik A.F., El-Khrisy E.E.-D.A., Dawidar A.-A.M., Bystrom A., Mabry T.J. (2002). A benzoquinone and flavonoids from *Cyperus alopecuroides*. Phytochemistry.

[13] Abdel-Razik A.F., Nassar M.I., El-Khrisy E.-D.A., Dawidar A.-A.M., Mabry T.J. (2005). New prenylflavans from *Cyperus conglomeratus*. Fitoterapia.

[14] Seabra R.M., Moreira M.M., Costa M.A.C., Paul M.I. (1995). 6,3′,4′-trihydroxy-4-methoxy-5-methylaurone from *Cyperus capitatus*. Phytochemistry.

[15] Seabra R.M., Silva A.M.S., Andrade P.B., Manuela Moreira M. (1998). Methylaurones from *Cyperus capitatus*. Phytochemistry.

[16] Xu Y., Zhang H.-W., Yu C.-Y., Lu Y., Chang Y., Zou Z.-M. (2008). Norcyperone, a novel skeleton norsesquiterpene from *Cyperus rotundus* L.. Molecules.

[17] Gamal M., Hani K.M., Sameh E.S., Sabrin I.R. (2015). A review: Compounds isolated from *Cyperus* species (Part I): Phenolics and nitrogenous. Int. J. Pharmacogn. Phytochem. Res..

[18] Hisham A., Rameshkumar K.B., Sherwani N., Al-Saidi S., Al-Kindy S. (2012). The composition and antimicrobial activities of *Cyperus conglomeratus*, *Desmos chinensis* var. *lawii* and *Cyathocalyx zeylanicus* essential oils. Nat. Prod. Commun..

[19] Al-Hazmi G.H., Awaad A.S., Alothman M.R., Alqasoumi S.I. (2018). Anticandidal activity of the extract and compounds isolated from *Cyperus conglomertus* Rottb. Saudi Pharm. J..

[20] Konturek P.C., Duda A., Brzozowski T., Konturek S., Kwiecien S., Drozdowicz D., Pajdo R., Meixner H., Hahn E. (2000). Activation of genes for superoxide dismutase, interleukin-1ß, tumor necrosis factor-a, and intercellular adhesion molecule-1 during healing of ischemia-reperfusion-induced gastric injury. Scand. J. Gastroenterol..

[21] Kwiecien S., Brzozowski T., Konturek S. (2002). Effects of reactive oxygen species action on gastric mucosa in various models of mucosal injury. J. Physiol. Pharmacol..

[22] Elshamy A.I., Farrag A.H., Mohamed S.H., Ali N.A., Mohamed T.A., Menshawy M., Zaglool A., Efferth T., Hegazy M.-E. (2020). Gastroprotective effects of ursolic acid isolated from *Ochrosia elliptica* on ethanol-induced gastric ulcer in rats. Med. Chem. Res..

[23] Elkady W.M., Ayoub I.M., Abdel-Mottaleb Y., ElShafie M.F., Wink M. (2020). *Euryops pectinatus* L. Flower extract inhibits p-glycoprotein and reverses multi-drug resistance in cancer cells: A mechanistic study. Molecules.

[24] Fang N., Yu S., Prior R.L. (2002). LC/MS/MS characterization of phenolic constituents in dried plums. J. Agric. Food Chem..

[25] Faheem S.A., Saeed N.M., El-Naga R.N., Ayoub I.M., Azab S.S. (2020). Hepatoprotective effect of cranberry nutraceutical extract in non-alcoholic fatty liver model in rats: Impact on insulin resistance and Nrf-2 expression. Front. Pharmacol..

[26] Lin L.-Z., Sun J., Chen P., Monagas M.J., Harnly J.M. (2014). UHPLC-PDA-ESI/HRMSn profiling method to identify and quantify oligomeric proanthocyanidins in plant products. J. Agric. Food Chem..

[27] Ren Z., Nie B., Liu T., Yuan F., Feng F., Zhang Y., Zhou W., Xu X., Yao M., Zhang F. (2016). Simultaneous determination of coumarin and its derivatives in tobacco products by Liquid Chromatography-Tandem Mass Spectrometry. Molecules.

[28] Farag M.A., Sharaf Eldin M.G., Kassem H., Abou el Fetouh M. (2013). Metabolome classification of *Brassica napus* L. organs via UPLC–QTOF–PDA–MS and their anti-oxidant potential. Phytochem. Anal..

[29] Thomson R. (2012). Naturally Occurring Quinones.

[30] Yang M., Wang W., Sun J., Zhao Y., Liu Y., Liang H., Guo D.A. (2007). Characterization of phenolic compounds in the crude extract of *Hedysarum multijugum* by high-performance liquid chromatography with electrospray ionization tandem mass spectrometry. Rapid Commun. Mass Spectrom..

[31] Morikawa T., Xu F., Matsuda H., Yoshikawa M. (2010). Structures of novel norstilbene dimer, longusone A, and three new stilbene dimers, longusols A, B, and C, with antiallergic and radical scavenging activities from Egyptian natural medicine *Cyperus longus*. Chem. Pharm. Bull..

[32] Farag M.A., Khattab A.R., Maamoun A.A., Kropf M., Heiss A.G. (2019). UPLC-MS metabolome based classification of *Lupinus* and *Lens* seeds: A prospect for phyto-equivalency of its different accessions. Food Res. Int..

[33] Cheng C., Chen Y., Ye Q., Liang Y., He X., Zhou Z., Feng Z. (2014). A new isoflavonoid from the rhizomes of *Cyperus rotundus*. Asian J. Chem..

[34] Abdel-Mogib M., Basaif S., Ezmirly S. (2000). Two novel flavans from Cyperus conglomeratus. Pharmazie.

[35] Farag M.A., Ezzat S.M., Salama M.M., Tadros M.G. (2016). Anti-acetylcholinesterase potential and metabolome classification of 4 *Ocimum* species as determined via UPLC/qTOF/MS and chemometric tools. J. Pharm. Biomed. Anal..

[36] Abu-Reidah I.M., Contreras M.M., Arraez-Roman D., Segura-Carretero A., Fernandez-Gutierrez A. (2013). Reversed-phase ultra-high-performance liquid chromatography coupled to electrospray ionization-quadrupole-time-of-flight mass spectrometry as a powerful tool for metabolic profiling of vegetables: *Lactuca sativa* as an example of its application. J. Chromatogr. A.

[37] Rabelo A.S., Serafini M.R., Rabelo T.K., de Melo M.G.D., da Silva Prado D., Gelain D.P., Moreira J.C.F., dos Santos Bezerra M., da Silva T.B., Costa E.V. (2014). Chemical composition, antinociceptive, anti-inflammatory and redox properties in vitro of the essential oil from *Remirea maritima* Aubl. (Cyperaceae). BMC Complementary Altern. Med..

[38] Farag M.A., Porzel A., Schmidt J., Wessjohann L.A. (2012). Metabolite profiling and fingerprinting of commercial cultivars of *Humulus lupulus* L. (hop): A comparison of MS and NMR methods in metabolomics. Metabolomics.

[39] Shen J., Wei J., Li L., Ouyang H., Chang Y., Chen X., He J. (2018). “Development of a HPLC-MS/MS method to determine 11 bioactive compounds in Tongmai Yangxin Pill and application to a pharmacokinetic study in rats. Evid. Based Compl. Alt..

[40] Amesty Á., Burgueño-Tapia E., Joseph-Nathan P., Ravelo Á.G., Estévez-Braun A. (2011). Benzodihydrofurans from *Cyperus teneriffae*. J. Nat. Prod..

[41] Guo J., Liu D., Nikolic D., Zhu D., Pezzuto J.M., van Breemen R.B. (2008). In vitro metabolism of isoliquiritigenin by human liver microsomes. Drug Metab. Dispos..

[42] Alanís R.M., Kennedy J.F., Yannai S. (2004). Dictionary of Food Compounds with CD-Rom: Additives. Flavors, and Ingredients.

[43] Appleyard C., McCafferty D., Tigley A., Swain M., Wallace J. (1996). Tumor necrosis factor mediation of NSAID-induced gastric damage: Role of leukocyte adherence. Am. J. Physiol. Gastrointest. Liver Physiol..

[44] Santucci L., Fiorucci S., Di Matteo F.M., Morelli A. (1995). Role of tumor necrosis factor α release and leukocyte margination in indomethacin-induced gastric injury in rats. Gastroenterology.

[45] Wallace J.L. (2008). Prostaglandins, NSAIDs, and gastric mucosal protection: Why doesn’t the stomach digest itself?. Physiol. Rev..

[46] Wang L., Zhou Y., Peng J., Zhang Z., Jiang D.-J., Li Y.-J. (2008). Role of endogenous nitric oxide synthase inhibitor in gastric mucosal injury. Can. J. Physiol. Pharm..

[47] Mota C., Freitas R., Athayde M., Boligon A., Augusti P., Somacal S., Rocha M., Bauermann L. (2011). Effect of *Vernonia cognata* on oxidative damage induced by ethanol in rats. Hum. Exp. Toxicol..

[48] Ganguly K., Maity P., Reiter R.J., Swarnakar S. (2005). Effect of melatonin on secreted and induced matrix metalloproteinase-9 and-2 activity during prevention of indomethacin-induced gastric ulcer. J. Pineal Res..

[49] Amagase K., Yokota M., Tsukimi Y., Okabe S. (2003). Characterization of “unhealed gastric ulcers” produced with chronic exposure of acetic acid ulcers to indomethacin in rats. J. Physiol. Pharmacol..

[50] Potrich F.B., Allemand A., da Silva L.M., dos Santos A.C., Baggio C.H., Freitas C.S., Mendes D.A.G.B., Andre E., de Paula Werner M.F., Marques M.C.A. (2010). Antiulcerogenic activity of hydroalcoholic extract of *Achillea millefolium* L.: Involvement of the antioxidant system. J. Ethnopharmacol..

[51] Tsukimi Y., Okabe S. (2001). Recent advances in gastrointestinal pathophysiology: Role of heat shock proteins in mucosal defense and ulcer healing. Biol. Pharm. Bull..

[52] Salga M.S., Ali H.M., Abdulla M.A., Abdelwahab S.I. (2012). Gastroprotective activity and mechanism of novel dichlorido-zinc (II)-4-(2-(5-methoxybenzylideneamino) ethyl) piperazin-1-iumphenolate complex on ethanol-induced gastric ulceration. Chem. Biol. Interact..

[53] Choi J.-i., Raghavendran H.R.B., Sung N.-Y., Kim J.-H., Chun B.S., Ahn D.H., Choi H.-S., Kang K.-W., Lee J.-W. (2010). Effect of fucoidan on aspirin-induced stomach ulceration in rats. Chem. Biol. Interact..

[54] Diamond J., Pesek I. (1991). Glomerular tumor necrosis factor and interleukin 1 during acute aminonucleoside nephrosis. An immunohistochemical study. Lab. Investig..

[55] Faubion W.A., Gores G.J. (1999). Death receptors in liver biology and pathobiology. Hepatology.

[56] Yadav S.K., Adhikary B., Chand S., Maity B., Bandyopadhyay S.K., Chattopadhyay S. (2012). Molecular mechanism of indomethacin-induced gastropathy. Free Radic. Biol. Med..

[57] Brinchmann M.F., Patel D.M., Iversen M.H. (2018). The role of galectins as modulators of metabolism and inflammation. Mediat. Inflamm..

[58] Johannes L., Jacob R., Leffler H. (2018). Galectins at a glance. J. Cell Sci..

[59] DeRoo E.P., Wrobleski S.K., Shea E.M., Al-Khalil R.K., Hawley A.E., Henke P.K., Myers D.D., Wakefield T.W., Diaz J.A. (2015). The role of galectin-3 and galectin-3–binding protein in venous thrombosis. Blood.

[60] Sciacchitano S., Lavra L., Morgante A., Ulivieri A., Magi F., De Francesco G.P., Bellotti C., Salehi L.B., Ricci A. (2018). Galectin-3: One molecule for an alphabet of diseases, from A to Z. Int. J. Mol. Sci..

[61] Uhlén M., Fagerberg L., Hallström B.M., Lindskog C., Oksvold P., Mardinoglu A., Sivertsson Å., Kampf C., Sjöstedt E., Asplund A. (2015). Tissue-based map of the human proteome. Science.

[62] Sidahmed H.M.A., Azizan A.H.S., Mohan S., Abdulla M.A., Abdelwahab S.I., Taha M.M.E., Hadi A.H.A., Ketuly K.A., Hashim N.M., Loke M.F. (2013). Gastroprotective effect of desmosdumotin C isolated from Mitrella kentii against ethanol-induced gastric mucosal hemorrhage in rats: Possible involvement of glutathione, heat-shock protein-70, sulfhydryl compounds, nitric oxide, and anti-Helicobacter pylori activity. BMC Complem. Altern. Med..

[63] Park S.W., Oh T.Y., Kim Y.S., Sim H., Park S.J., Jang E.J., Park J.S., Baik H.W., Hahm K.B. (2008). *Artemisia asiatica* extracts protect against ethanol-induced injury in gastric mucosa of rats. J. Gastroenterol. Hepatol..

[64] Silva M.I., Moura M.A., de Aquino Neto M.R., da Rocha T.A., Rocha N.F., de Carvalho A.M., Macêdo D.S., Vasconcelos S.M., de Sousa D.P., Viana G.S. (2009). Gastroprotective activity of isopulegol on experimentally induced gastric lesions in mice: Investigation of possible mechanisms of action. N-S. Arch. Pharmacol..

[65] Sannomiya M., Fonseca V.B., Da Silva M., Rocha L., Dos Santos L., Hiruma-Lima C., Brito A.S., Vilegas W. (2005). Flavonoids and antiulcerogenic activity from *Byrsonima crassa* leaves extracts. J. Ethnopharmacol..

[66] Algebali M., Menze E.T., Ayoub I.M., Tadros M., Esmat A. (2020). Macro and microscopic gastroprotective effects of grape seed extract on the gastric ulcer experimentally induced by alcohol. Arch. Pharm. Sci. Ain Shams Univ..

[67] Bhoumik D., Masresha B., Mallik A. (2017). Antiulcer properties of herbal drugs: A review. Int. J. Biomed. Res..

[68] Golbabapour S., Gwaram N.S., Hassandarvish P., Hajrezaie M., Kamalidehghan B., Abdulla M.A., Ali H.M., Hadi A.H.A., Majid N.A. (2013). Gastroprotection studies of Schiff base zinc (II) derivative complex against acute superficial hemorrhagic mucosal lesions in rats. PLoS ONE.

[69] Hajrezaie M., Golbabapour S., Hassandarvish P., Gwaram N.S., Hadi A.H.A., Ali H.M., Majid N., Abdulla M.A. (2012). Acute toxicity and gastroprotection studies of a new schiff base derived copper (II) complex against ethanol-induced acute gastric lesions in rats. PLoS ONE.

[70] Chauhan I., Agrawal S., Goel R.K. (2018). Status of inflammatory markers and growth factor in gastric ulcer protective effects of *Punica granatum* L. peel extract in rat. Natl. J. Physiol. Pharm. Pharmacol..

[71] Bauer H., Jung T., Tsikas D., Stichtenoth D., FRÖLICH C., Neumann C. (1997). Nitric oxide inhibits the secretion of T-helper 1-and T-helper 2-associated cytokines in activated human T cells ‘pa. Immunology.

[72] Batista L., De Morais L.G., De Almeida A., Magri L., Calvo T., Ferreira A., Pellizzon C., Hiruma-Lima C., Vilegas W., Sano P. (2015). Ulcer healing and mechanism(s) of action involved in the gastroprotective activity of fractions obtained from *Syngonanthus arthrotrichus* and *Syngonanthus bisulcatus*. BMC Complementary Altern. Med..

[73] Zakaria Z.A., Balan T., Suppaiah V., Ahmad S., Jamaludin F. (2014). Mechanism+(s) of action involved in the gastroprotective activity of *Muntingia calabura*. J. Ethnopharmacol..

[74] Abdelwahab S.I., Mohan S., Abdulla M.A., Sukari M.A., Abdul A.B., Taha M.M.E., Syam S., Ahmad S., Lee K.-H. (2011). The methanolic extract of *Boesenbergia rotunda* (L.) Mansf. and its major compound pinostrobin induces anti-ulcerogenic property in vivo: Possible involvement of indirect antioxidant action. J. Ethnopharmacol..

[75] La Casa C., Villegas I., De La Lastra C.A., Motilva V., Calero M.M. (2000). Evidence for protective and antioxidant properties of rutin, a natural flavone, against ethanol induced gastric lesions. J. Ethnopharmacol..

[76] Martín M., De La Lastra C.A., Motilva V., La Casa C. (2000). Antiulcer and gastroprotective activity of flavonic compounds: Mechanisms involved. Stud. Nat. Prod. Chem..

[77] Sumbul S., Ahmad M.A., Mohd A., Mohd A. (2011). Role of phenolic compounds in peptic ulcer: An overview. J. Pharm. Bioallied Sci..

[78] Trautmann M., Peskar B.M., Peskar B.A. (1991). Aspirin-like drugs, ethanol-induced rat gastric injury and mueosal eicosanoid release. Eur. J. Pharmacol..

[79] Taofiq O., González-Paramás A.M., Barreiro M.F., Ferreira I.C.F.R. (2017). Hydroxycinnamic acids and their derivatives: Cosmeceutical significance, challenges and future perspectives, a review. Molecules.

[80] Alam M.A., Subhan N., Hossain H., Hossain M., Reza H.M., Rahman M.M., Ullah M.O. (2016). Hydroxycinnamic acid derivatives: A potential class of natural compounds for the management of lipid metabolism and obesity. Nutr. Metab..

[81] Jang S.-A., Park D.W., Kwon J.E., Song H.S., Park B., Jeon H., Sohn E.-H., Koo H.J., Kang S.C. (2017). Quinic acid inhibits vascular inflammation in TNF-*α*-stimulated vascular smooth muscle cells. Biomed. Pharmacother..

[82] Abdel Motaal A., Ezzat S.M., Tadros M.G., El-Askary H.I. (2016). In vivo anti-inflammatory activity of caffeoylquinic acid derivatives from *Solidago virgaurea* in rats. Pharm. Biol..

[83] Borrelli F., Izzo A.A. (2000). The plant kingdom as a source of anti-ulcer remedies. Phytother. Res..

[84] Iqbal M., Verpoorte R., Korthout H.A., Mustafa N.R. (2013). Phytochemicals as a potential source for TNF-*α* inhibitors. Phytochem. Rev..

[85] Maamoun A.A., El-akkad R.H., Farag M.A. (2019). Mapping metabolome changes in *Luffa aegyptiaca* Mill fruits at different maturation stages via MS-based metabolomics and chemometrics. J. Adv. Res..

[86] Bancroft J.D., Gamble M. (2008). Theory and Practice of Histological Techniques.

[87] Nordin N., Salama S.M., Golbabapour S., Hajrezaie M., Hassandarvish P., Kamalidehghan B., Majid N.A., Hashim N.M., Omar H., Fadaienasab M. (2014). Anti-ulcerogenic effect of methanolic extracts from *Enicosanthellum pulchrum* (King) Heusden against ethanol-induced acute gastric lesion in animal models. PLoS ONE.

