# Supplementary material for: UPLC-qTOF-MS Phytochemical Profile and Antiulcer Potential of Cyperus conglomeratus Rottb. Alcoholic Extract
Source: Molecules. 2020 Sep 15;25(18):4234. doi: 10.3390/molecules25184234 (PMC7570889; doi:10.3390/molecules25184234)
Supplement: Supplementary file 1 [file molecules-25-04234-s001.pdf]

molecules

MDPI

Supplementary Materials

UPLC-qTOF-MS phytochemical profile and antiulcer potential of *Cyperus conglomeratus* Rottb. alcoholic extract

Abdelsamed I. Elshamy[1*], Abdel Razik H. Farrag[2], Iriny M. Ayoub[3], Karam A. Mahdy[4], Rehab F. Taher[1], Abd El-Nasser G. El Gendy[5], Tarik A. Mohamed[6], Salim S. Al-Rejaie[7], Yasser A. El-Amier[8], Ahmed M. Abd-ElGawad[8,9*] and Mohamed A. Farag[10,11]

[1] Department of Natural Compounds Chemistry, National Research Center, 33 El Bohouth St., Dokki, Giza 12622, Egypt; elshamynrc@yahoo.com; rehabfikrytaher@gmail.com
[2] Pathology Department; National Research Centre, 33 El Bohouth St., Dokki, Giza 12622, Egypt; abdelrazik2000@gmail.com
[3] Pharmacognosy Department, Faculty of Pharmacy, Ain Shams University, Cairo 11566, Egypt; irinyayoub@pharma.asu.edu.eg
[4] Medical Biochemistry Department; National Research Centre, 33 El Bohouth St., Dokki, Giza 12622, Egypt; karammahdy64@gmail.com
[5] Medicinal and Aromatic Plants Research Department, National Research Centre, 33 El Bohouth St., Dokki, Giza 12622, Egypt; aggundy_5@yahoo.com
[6] Chemistry of Medicinal Plants Department, National Research Centre, 33 El-Bohouth St., Dokki, Giza, 12622, Egypt; tarik.nrc83@yahoo.com
[7] Department of Pharmacology & Toxicology, College of Pharmacy, King Saud University, Riyadh 11451, Saudi Arabia; rejaie@ksu.edu.sa
[8] Department of Botany, Faculty of Science, Mansoura University, Mansoura 35516, Egypt; dgawad84@mans.edu.eg, yasran@mans.edu.eg
[9] Plant Production Department, College of Food & Agriculture Sciences, King Saud University, P.O. Box 2460, Riyadh 11451, Saudi Arabia; aibrahim2@ksu.edu.sa
[10] Pharmacognosy Department, College of Pharmacy, Cairo University, Kasr el Aini St., P.B. 11562, Cairo, Egypt; mfarag73@yahoo.com
[11] Chemistry Department, School of Sciences & Engineering, The American University in Cairo, New Cairo 11835, Egypt;

Correspondence: elshamynrc@yahoo.com; +20-1005525108 (A.I.E.), aibrahim2@ksu.edu.sa; Tel.: +966-562680864 (A.M.A.-E.)

Received: date; Accepted: date; Published: date

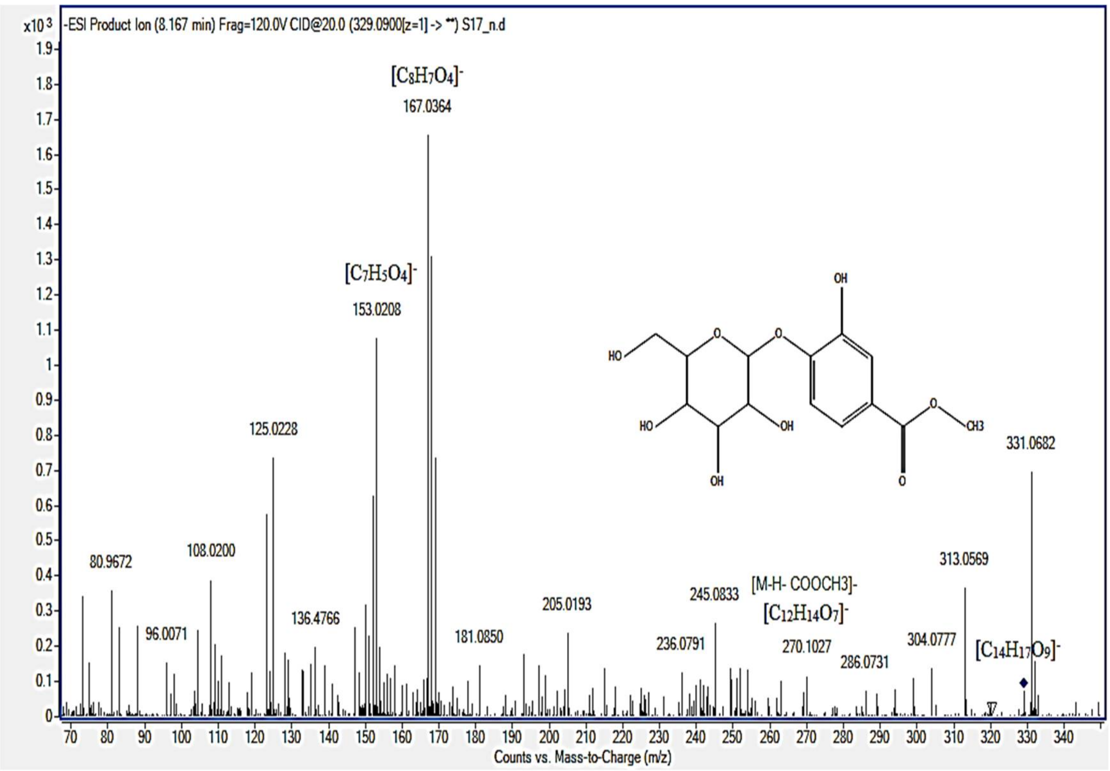

Figure S1. Negative ESI tandem mass spectra of dihydroxy benzoic acid methyl ester hexoside (peak 21)

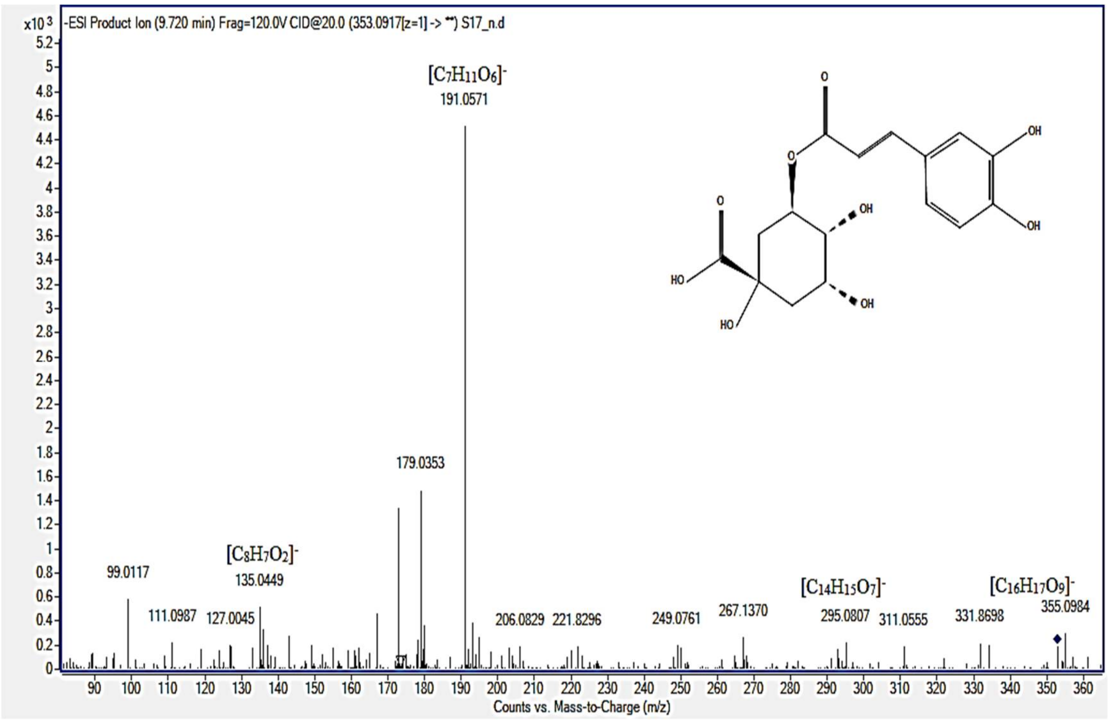

Figure S2. Negative ESI tandem mass spectra of *O*-caffeoylquinic acid (peak 34)

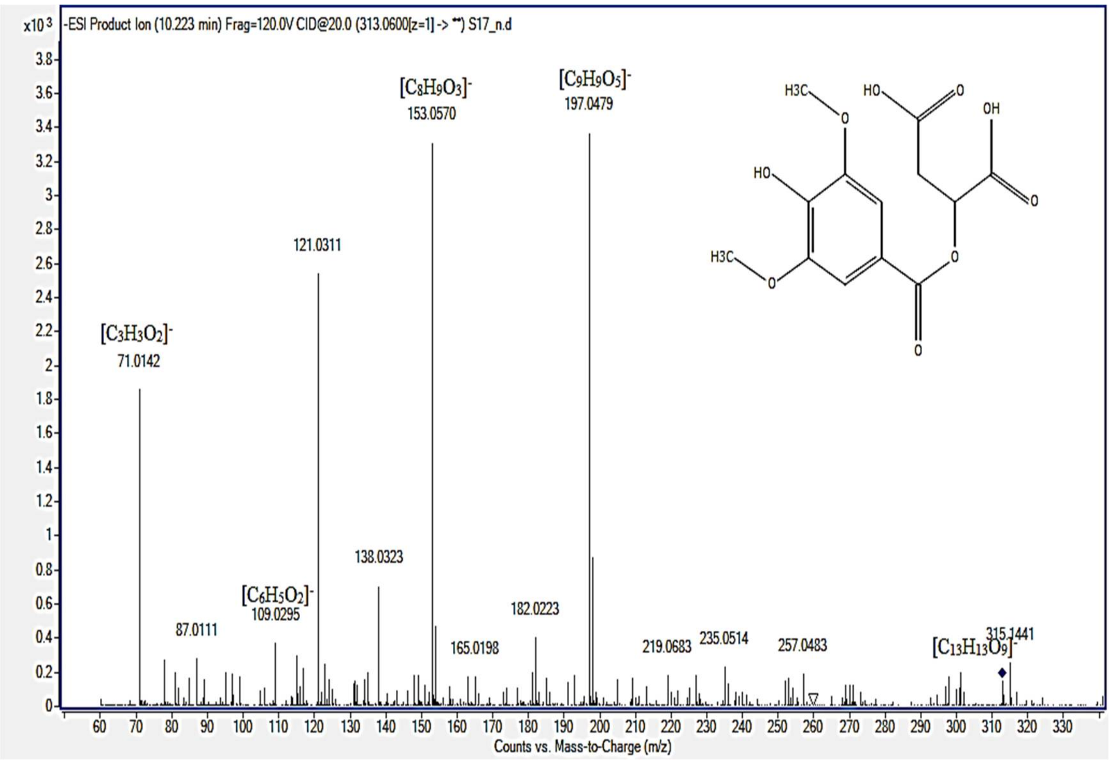

Figure S3. Negative ESI tandem mass spectra of syringoylmalic acid (peak 39)

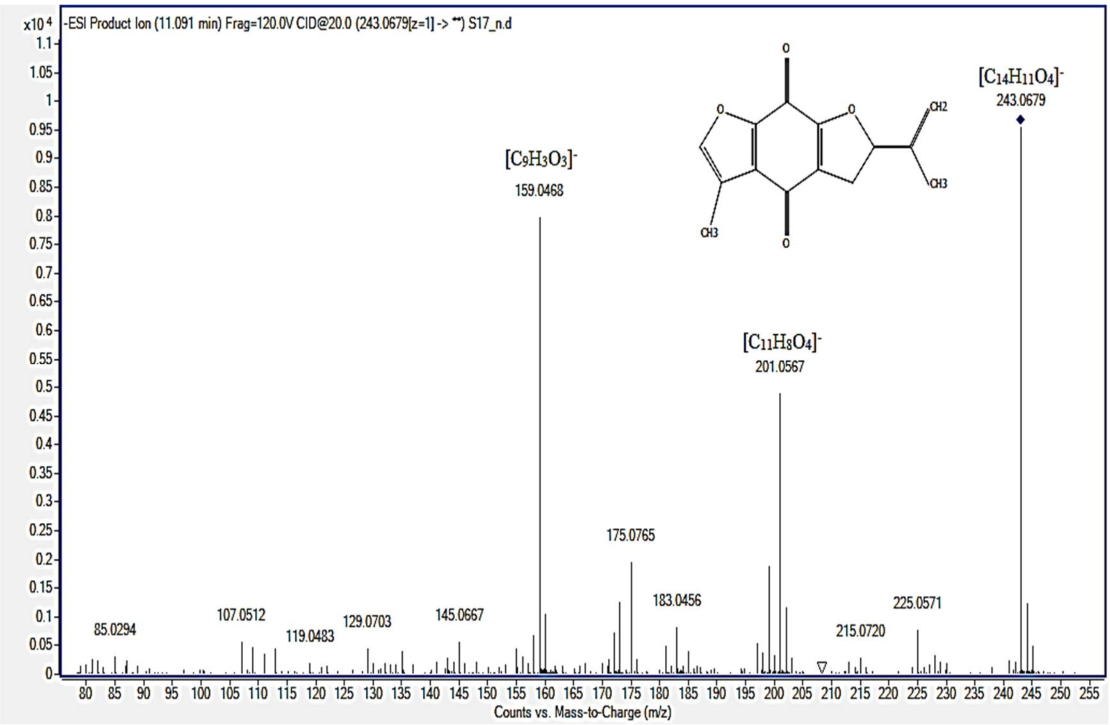

Figure S4. Negative ESI tandem mass spectra of dihydrocyperaquinone (peak 48)

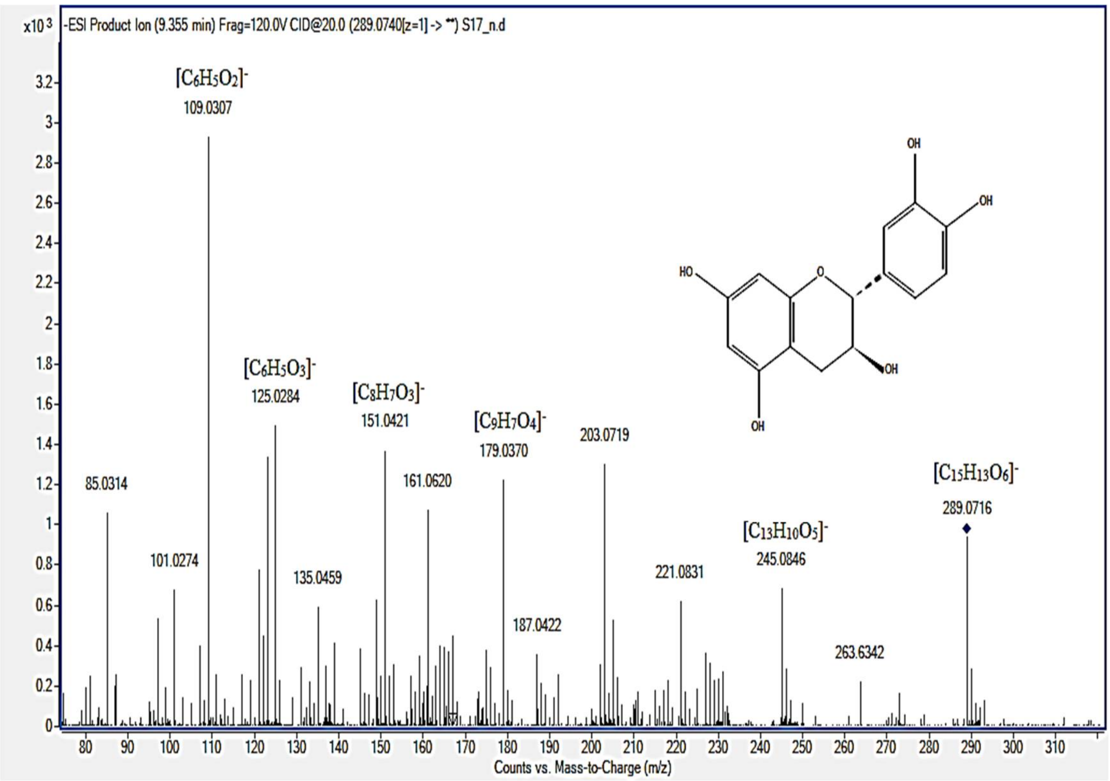

Figure S5. Negative ESI tandem mass spectra of (epi)catechin (peak 30)

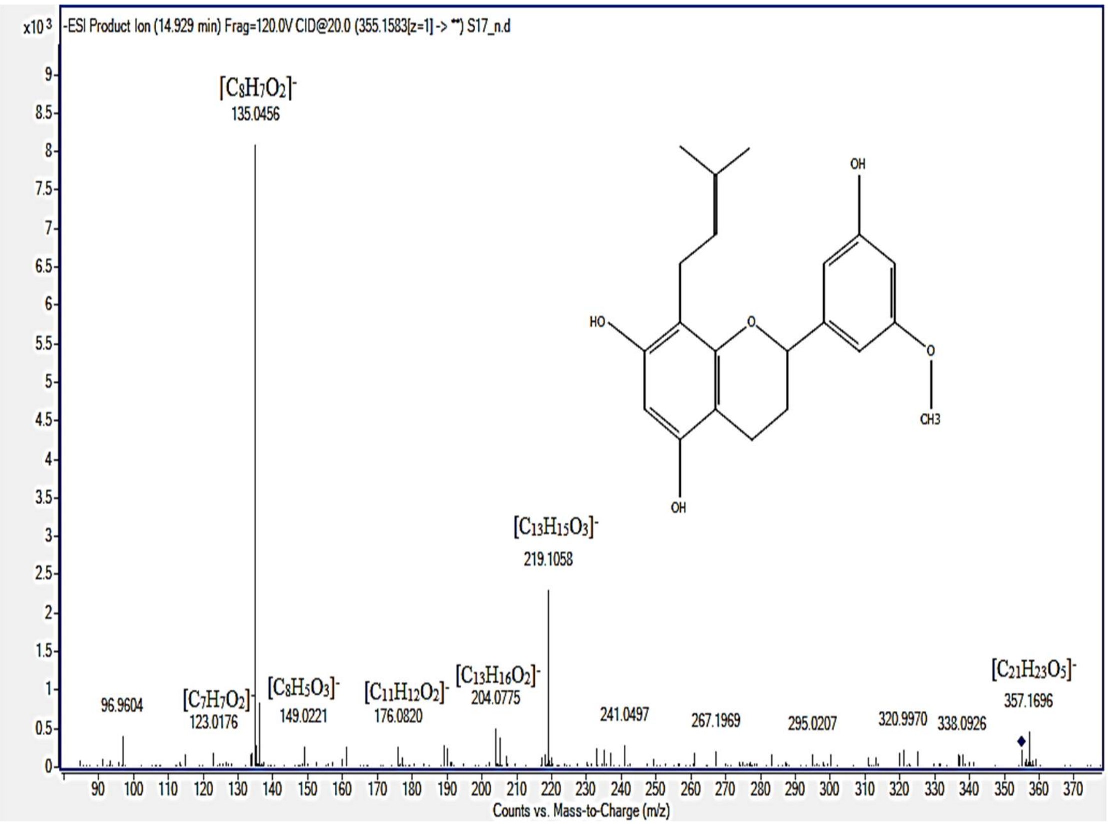

Figure S6. Negative ESI tandem mass spectra of trihydroxy-methoxy-prenylflavan (peak 70)

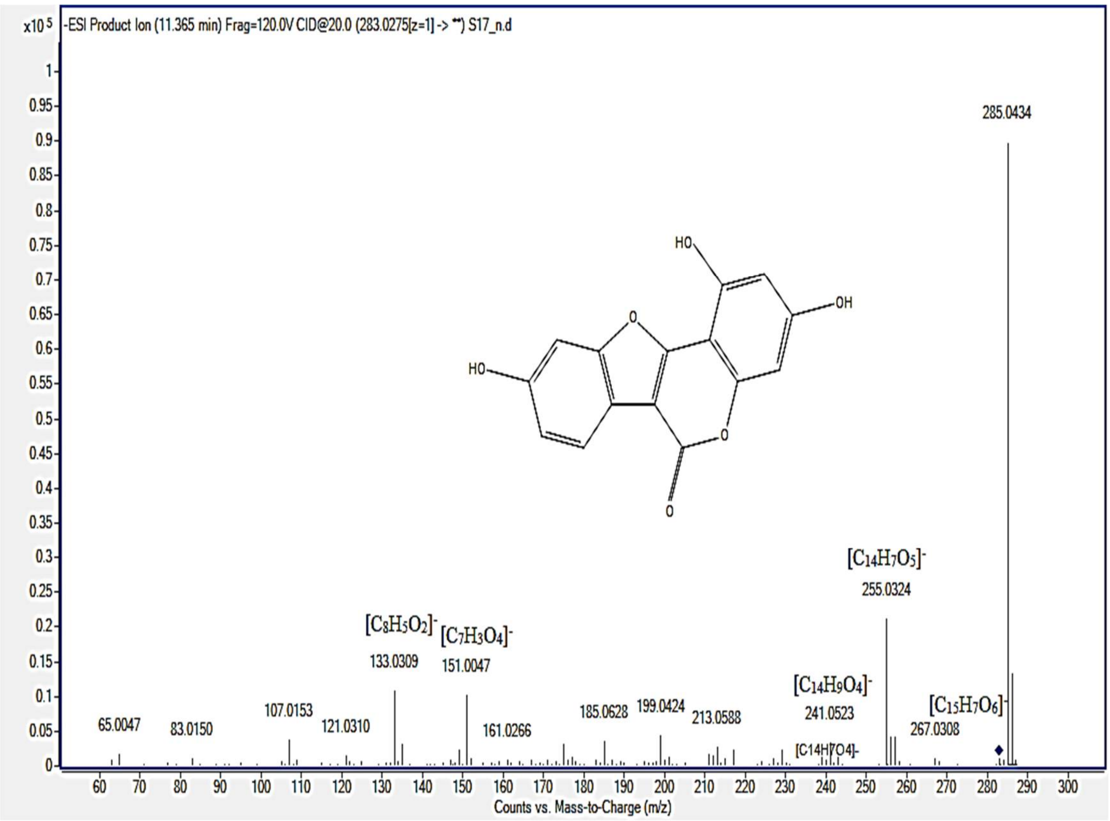

Figure S7. Negative ESI tandem mass spectra of trihydroxycoumestan (peak 50)

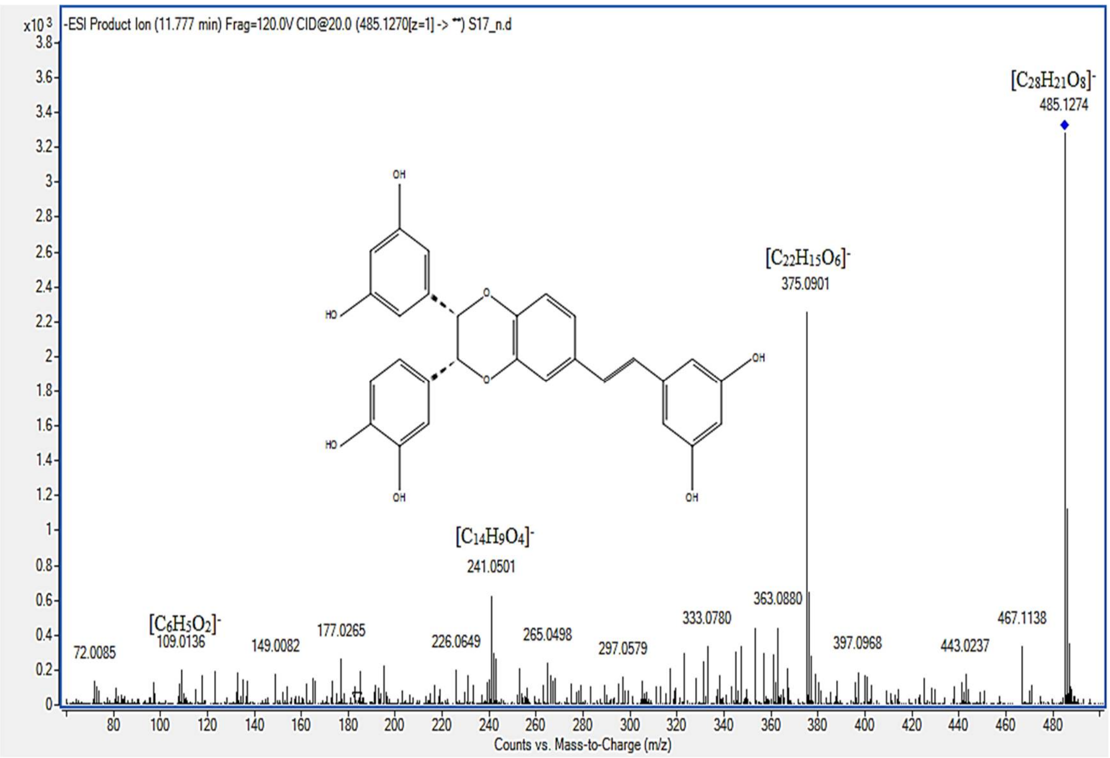

Figure S8. Negative ESI tandem mass spectra of Longusol C (peak 53)

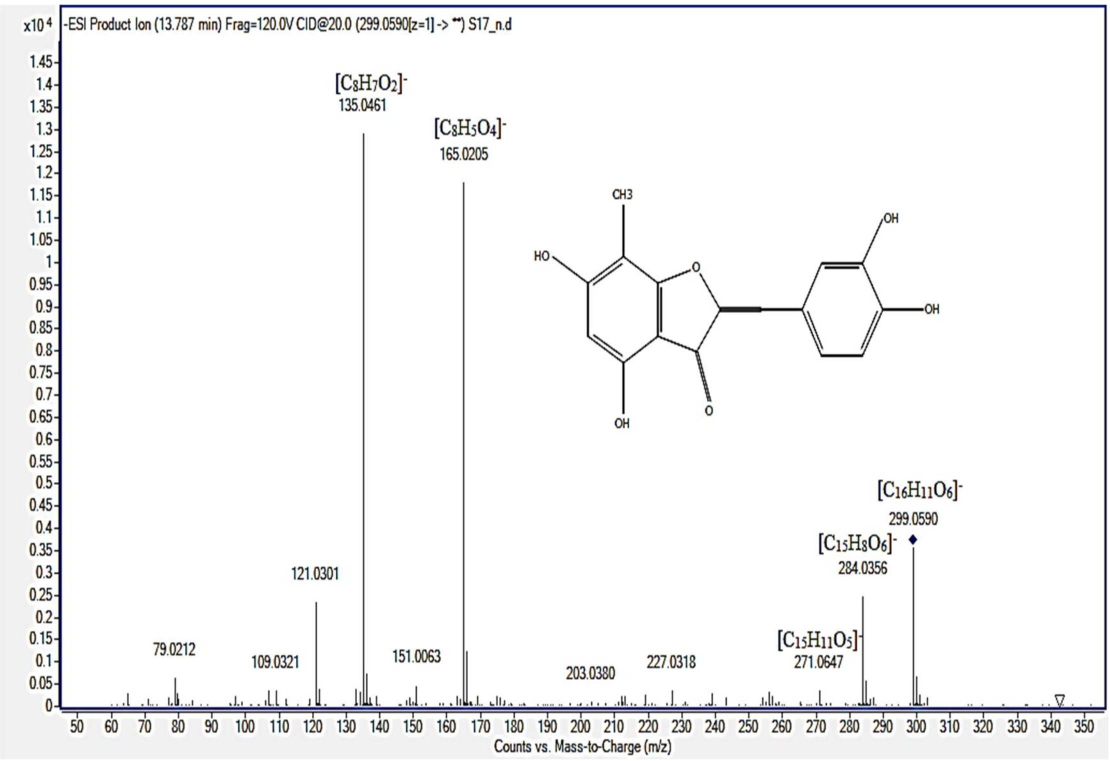

Figure S9. Negative ESI tandem mass spectra of tetrahydroxy methyl aurone (peak 66)

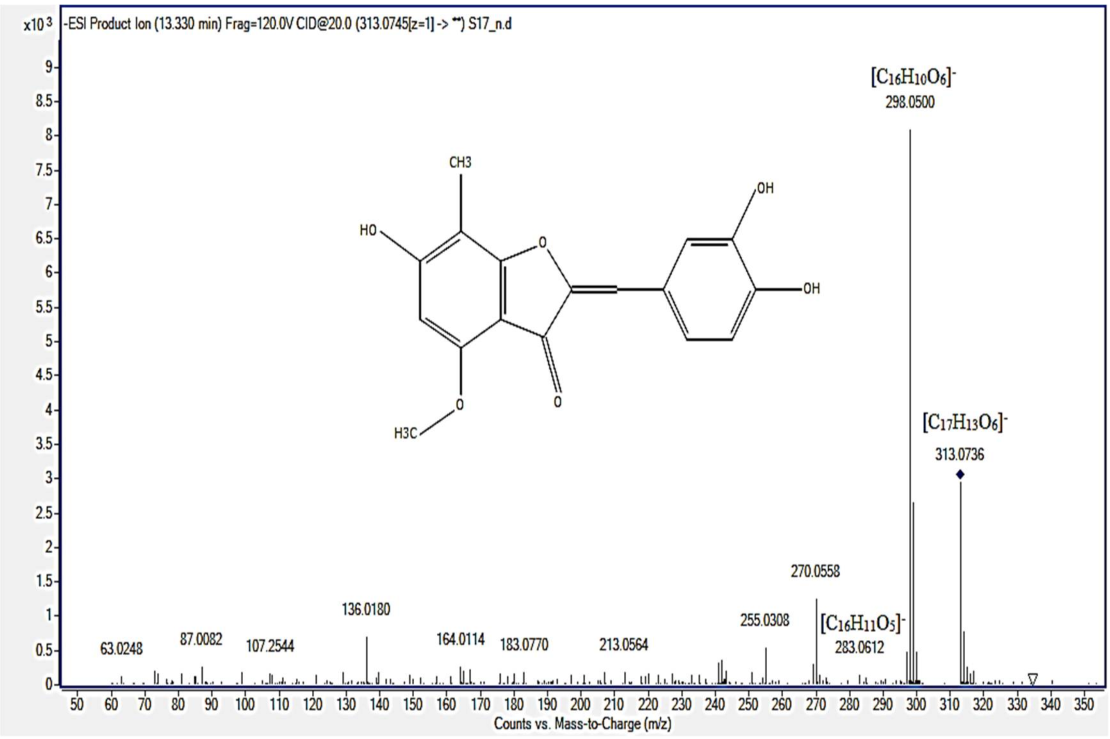

Figure S10. Negative ESI tandem mass spectra of trihydroxy-methoxy methyl aurone (peak 64)

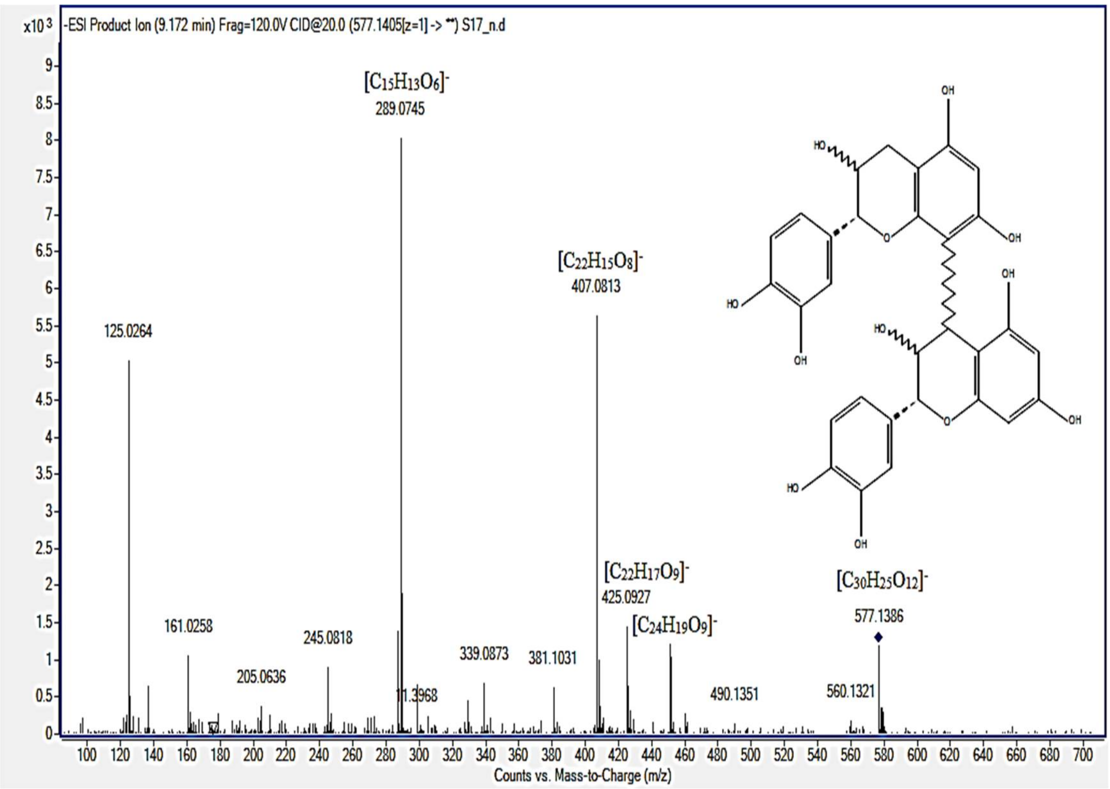

Figure S11. Negative ESI tandem mass spectra of procyanidin B dimer (peak 26)

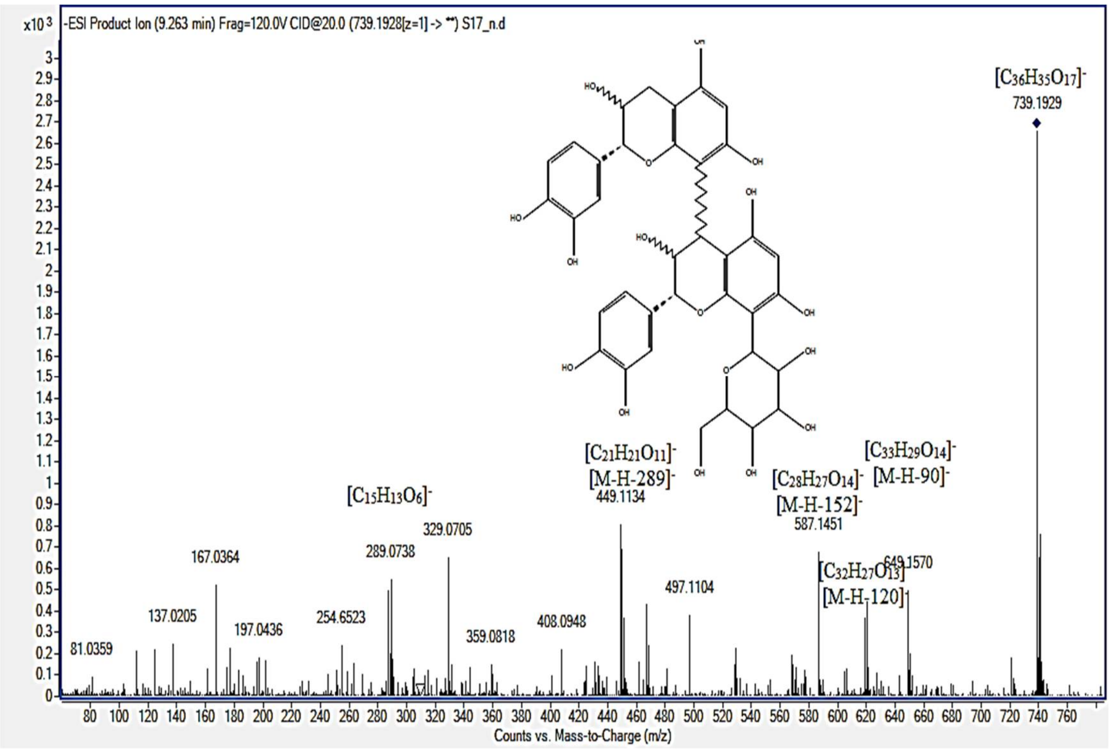

Figure S12. Negative ESI tandem mass spectra of *C*-hexosyl procyanidin B dimer (peak 28)
